# High‐Throughput Screening of Bicationic Redox Materials for Chemical Looping Ammonia Synthesis

**DOI:** 10.1002/advs.202202811

**Published:** 2022-07-24

**Authors:** Jiaxin Fan, Wenxian Li, Sean Li, Jack Yang

**Affiliations:** ^1^ Materials and Manufacturing Futures Institute School of Material Science and Engineering University of New South Wales Sydney New South Wales 2052 Australia

**Keywords:** bicationic redox pairs, chemical looping ammonia synthesis, cooperative effect, high‐throughput materials screening, machine learning, reaction engineering

## Abstract

Ammonia recently has gained increasing attention as a carrier for the efficient and safe usage of hydrogen to further advance the hydrogen economy. However, there is a pressing need to develop new ammonia synthesis techniques to overcome the problem of intense energy consumption associated with the widely used Haber–Bosch process. Chemical looping ammonia synthesis (CLAS) is a promising approach to tackle this problem, but the ideal redox materials to drive these chemical looping processes are yet to be discovered. Here, by mining the well‐established MP database, the reaction free energies for CLAS involving 1699 bicationic inorganic redox pairs are screened to comprehensively investigate their potentials as efficient redox materials in four different CLAS schemes. A state‐of‐the‐art machine learning strategy is further deployed to significantly widen the chemical space for discovering the promising redox materials from more than half a million candidates. Most importantly, using the three‐step H_2_O‐CL as an example, a new metric is introduced to determine bicationic redox pairs that are “cooperatively enhanced” compared to their corresponding monocationic counterparts. It is found that bicationic compounds containing a combination of alkali/alkaline‐earth metals and transition metal (TM)/post‐TM/metalloid elements are compounds that are particularly promising in this respect.

## Graphical abstract

The reaction thermodynamics of more than half a million bicationic solids are computationally screened to systematically access their potential in driving chemical looping ammonia synthesis. It is found that many candidates with new chemical compositions can facilitate these processes spontaneously. A “cooperativity” metric is further proposed to provide critical insights on whether these complex solids outperform their corresponding monocationic counterparts.

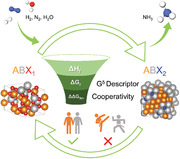

## 1 Introduction

Ammonia is a crucial industrial material that acts as a precursor for large‐scale manufacturing of products that are essential to sustain basic human life, including agricultural fertilizers, pharmaceutical drugs, explosives, and many others.^[^ ^1^, ^2^, ^3^, ^4^, ^5^, ^6^ ^]^ Recently, it is also considered as a promising medium to facilitate the global energy dispatch for hydrogen economy due to its easily liquefiable and less‐explosive nature. This is further stimulated by the existence of complete transport and storage infrastructures that have been developed for the ammonia industry over the past few decades.^[^ ^7^, ^8^, ^9^ ^]^

Currently, the most well‐established approach for manufacturing ammonia in industrial scale is the Haber–Bosch process, which catalytically converts nitrogen and hydrogen into ammonia at an elevated pressure (100–350 atm) and temperature (400–500 °C).^[^ ^10^ ^]^ This century‐old process is heavy in both energy consumption and greenhouse gas emissions, particularly when the energies are sourced from the combustion of fossil fuels. Therefore, significant efforts have been devoted to search for alternative green approaches in ammonia synthesis.^[^ ^11^, ^12^, ^13^, ^14^, ^15^, ^16^, ^17^, ^18^, ^19^, ^20^, ^21^, ^22^, ^23^, ^24^ ^]^ Within these approaches, chemical looping ammonia synthesis^[^ ^25^, ^26^, ^27^, ^28^ ^]^ (CLAS) is particularly attractive as it enables the synthetic process to be separately manipulated in sequential steps, providing more freedoms in selecting the most suitable redox materials to drive the chemical reactions.

**Figure 1:**
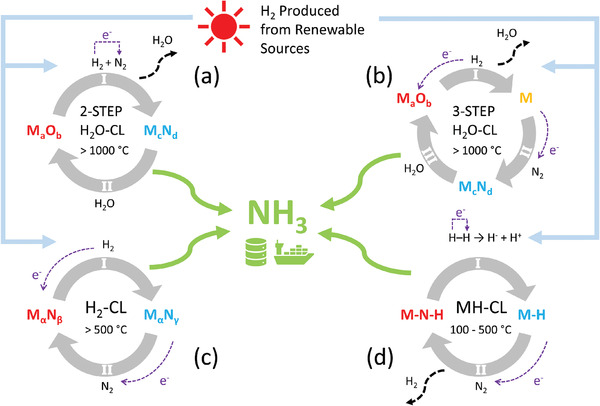
Schematic illustration of the state‐of‐the‐art CLAS mechanisms that involve the monocationic compounds (M–X) as the redox materials. a) two‐step and b) three‐step H_2_O‐CL. In these two chemical looping (CL) processes, metal nitrides are used as the nitrogen carriers, which are formed during the nitrogen harvest process (Rxn I in the two‐step loop and Rxn II in the three‐step loop, respectively). NH_3_ is generated when the nitrides are subsequently reacted with steam and simultaneously converted back to the corresponding oxides. c) H_2_‐CL, which is driven by either the conversion between two different nitrides or the formation and recovery of nitrogen vacancy (also known as the Mars‐van Krevelen mechanism^[^ ^29^, ^30^ ^]^). A nitrogen‐rich (N_rich_) metal nitride (M_α_N_β_) possessing cation in the high oxidation state is first reduced in H_2_ environment to generate NH_3_ (Rxn I). The nitrogen‐poor (N_poor_) nitride (M_α_N_γ_, 0 < γ < β) formed from the last step is then converted back to the corresponding N_rich_ phase by putting it in contact with N_2_ (Rxn II). d) MH‐CL, which has a similar looping mechanism as the H_2_‐CL loop. The main difference is in the redox materials, which MH‐CL involves the transformation between metal hydrides (M–H) and nitride‐hydrides (M–N–H). The sub‐reactions that involve H_2_ as reactant are labeled as Rxn I in all four CLs (a–d). More detailed chemical equations can be found in Section S1, Supporting Information.

The state‐of‐the‐art CLAS approaches can be categorized into four schemes where their looping mechanisms are depicted in **Figure** **1**.^[^ ^31^ ^]^ All CLAS processes involve the interconversions between active materials enabled by reacting them with different feedstocks. H_2_ is the most sustainable reducing agent to be used in CLAS for its low carbon footprint, especially when it is produced from renewable resources.^[^ ^32^, ^33^ ^]^ Figure 1a demonstrates a conventional two‐step chemical looping (CL) process that drives the NH_3_ formation through interconverting between a metal oxide and nitride redox pair (M_a_O_b_/M_c_N_d_). In this approach, N_2_ is fixed by M_a_O_b_ under the H_2_ environment (Rxn I) at high temperature (>1000 °C). NH_3_ then is generated from the subsequent hydrolysis of M_c_N_d_ (Rxn II, thus denoted as H_2_O‐CL).^[^ ^34^, ^35^ ^]^ Depending on the thermodynamic equilibria of the sub‐reactions, a three‐step variant (Figure 1b) of the H_2_O‐CL can be derived by splitting the nitrogen fixation step into an oxide reduction (Rxn I) and a metal nitridation (Rxn II) step.^[^ ^26^, ^36^, ^37^ ^]^ In this way, the formation of metallic intermediate could help to reduce the thermodynamic barrier for converting the oxide to nitride, thus improves the cyclic efficiency. In contrast to the previous two chemical loops, the H_2_‐CL (Figure 1c) purely loops between two nitrides with different nitrogen contents at a lower temperature (>500 °C). In H_2_‐CL, NH_3_ is generated from reacting H_2_ with the phase consisting of higher nitrogen content (M_α_N_β_) in Rxn I.^[^ ^25^, ^38^ ^]^ The reduced nitrogen‐deficient compound (M_α_N_γ_, 0 < γ < β) can then be used to capture N_2_ (Rxn II) thus regenerating M_α_N_β_ to complete the cycle. Similar conversion is also reported in the metal hydride (M–H) based system^[^ ^31^, ^39^, ^40^, ^41^ ^]^ shown in Figure 1d. Here, N_2_ is reduced by the anionic H^−^ in the metal hydride M–H (Rxn II), thus the loop is dubbed as MH‐CL. As a result, metal nitride‐hydride (M–N–H) is formed, which is then reacted with H_2_ in the hydrogenation step (Rxn I) to form NH_3_.

To date, the majority of studies on CLAS have been focusing on using monocationic compounds, that is, looping materials sharing one common cation (M–X), as the active redox materials. However, various challenges have been identified in association with the usage of this material system, including high reaction temperatures, sluggish kinetics, and low NH_3_ production rates.^[^ ^5^ ^]^ Recently, a few experimental attempts have been reported on the investigations of the bicationic compounds (with a general formula of M_1_–M_2_–X) as the active materials in CLAS.^[^ ^42^, ^43^, ^44^ ^]^ This sparks the search for suitable redox materials among compounds with more complex chemical compositions, as well as all different possible CLAS processes in which these materials may be applied. However, increasing the chemical complexity will also dramatically increase the number of candidature compounds, making the search with the traditional trial‐and‐error approach grossly inefficient. In this regard, high‐throughput computational material screenings have emerged to be an invaluable alternative in helping the community to expedite the process of property‐driven material development.^[^ ^25^, ^45^, ^46^, ^47^, ^48^ ^]^

In light of this, hereby, we extend the search of new redox materials for CLAS into a wider chemical space of bicationic compounds. In particular, the fast enumeration of the temperature‐dependent Gibbs reaction energies Δ*G*_r_(*T*) that involve 1699 M_1_–M_2_–X and 1647 M–X pairs are enabled by the usage of a machine‐learned Gibbs energy descriptor^[^ ^49^ ^]^ on materials recorded in the Materials Project (MP) database.^[^ ^50^ ^]^ Statistically, we have seen many more bicationic redox pairs that are energetically viable in driving three‐step H_2_O‐CL compared to two‐step H_2_O‐CL and H_2_‐CL with stronger thermodynamic driving forces. However, with only the compounds extracted from the MP database are not sufficient to complete all four different CLAS cycles. As such, using MH‐CL process as the example, we adopt the strategy of elemental substitution to generate 526,396 hypothetical compounds. Using a recently developed machine learning framework, their formation energies can be quickly predicted with almost negligible computational cost. With the further usage of convex hull stabilities as filtering criteria, additional 4698 potentially synthesizable redox pair candidates are attained to assess their potential for driving this particular process. The ability to predict and screen such a large set of hypothetical crystal structures, as a component of a single study, already represents a significant leap forward in what is achievable in modern crystal structure predictions.^[^ ^51^, ^52^, ^53^, ^54^ ^]^

Most importantly, to our best knowledge, no prior studies have been devoted to understand the possible thermodynamic advantages of using compounds with complex compositions in CLAS compared to those with simpler compositions. We will gain some critical insights to this question by considering a cooperativity metric calculated from the reaction energetics of three‐step H_2_O‐CL that are driven by the bicationic oxide/nitride redox pairs (M_1_–M_2_–O/N) with those driven by the monocationic redox pairs (M–O/N). More specifically, we reveal the possible “cooperative enhancement effect”^[^ ^55^ ^]^ being the key thermodynamic driving forces which favor M_1_–M_2_–O/N to be used as the redox materials. This is reflected by the reaction free energy of using M_1_–M_2_–O/N being even lower compared to the case of using a simple mixture of M_1_–O/N and M_2_–O/N. Such a cooperative effect is observable within certain groups of bicationic compounds, such as those composed with alkali/alkaline‐earth metals and first‐row transition metals. In principle, the cooperativity metric put forward in this work can also be used with reaction thermochemical data that is obtained experimentally, thus may open new ideas for hierarchically comparing the performances of complex solid‐state redox materials with the corresponding simpler counterparts.

## 2 Results and Discussions

### 2.1 Analysis of Temperature‐Dependent Thermodynamics of CLAS

#### 2.1.1 Construction of the Redox Pairs

As shown in **Table** **1**, the total numbers of bicationic compounds extracted from the MP database are significantly larger than the monocationic ones, particularly for oxides and nitrides. (Details of materials selection can be found in Section S2, Supporting Information) This seemingly suggests that it is likely to find much more viable redox pairs from compounds with complex chemical compositions. However, the number of redox pairs that can be formed among the available compounds are significantly less than the number of compounds that have been extracted from the MP database, which is limited by the stoichiometric constraint imposed in the chemical reactions (Equations (1)–(6) in Experimental Section). For example, CoMoO_4_ can only be paired with Co_3_Mo_3_N but not with Co_2_Mo_3_N or Co_3_W_3_N in the H_2_O‐CL. The most extreme case is the MH‐CL, whereby only one pair of redox materials (Li_2_MgH_4_ and Li_2_Mg(NH)_2_) was found to be able to satisfy the corresponding stoichiometric constraint. As such, in this section, we will focus our discussions on the temperature‐dependent reaction thermodynamics for CL processes a–c shown in Figure 1. The limitations in analyzing the MH‐CL process will be overcome by generating more hypothetical bicationic hydrides (M_1_–M_2_–H) and nitride‐hydrides (M_1_–M_2_–N–H), which will be discussed with more details in the later section.

**Table 1:** Numbers of crystalline solids in Materials Project database categorized by the anion groups as well as the constructed redox pairs based on the looping mechanisms demonstrated in Figure 1

System	Number of compounds				Number of pairs		
	Oxide	Nitride	Hydride	Nitride‐hydride	H_2_O‐CL	H_2_‐CL	MH‐CL
M–X	461	244	175	65	1174	473	136
M_1_–M_2_–X	9087	2378	394	111	1526	173	1

**Figure 2:**
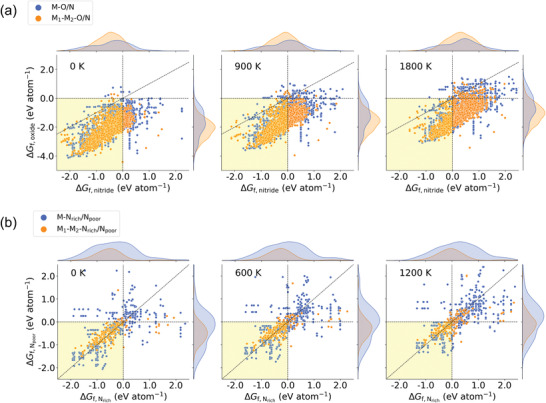
Comparison of the Gibbs formation energies (Δ*G*_f_(*T*)) of M–X and M_1_–M_2_–X pairs with respect to rising temperature. a) X = O and N, b) X = N_rich_ and N_poor_, which correspond to metal nitrides with higher/lower nitrogen contents, respectively. Redox pairs that are made from monocationic/bicationic compounds are colored in blue/orange markers respectively. The yellow‐shaded areas indicate that both solids in the active pairs have negative Gibbs formation energies meaning they are more stable and less likely to be decomposed at the temperature(s) considered.

Based on the redox pairs collated from the materials existing in the MP database, we first compare the temperature‐dependent Gibbs formation energies (Δ*G*_f_(*T*)) between the compounds in each redox pair, which is an important property that will determine the suitability for the redox pairs to drive specific CL reactions. **Figure** **2**a compares the Δ*G*_f_(*T*) for M_1_–M_2_–O/N and M–O/N calculated using the Gibbs energy descriptor (Equations 7‐9). The Δ*G*_f_(*T*) are calculated up to 1800 K to cover the high operation temperature for reducing oxides in H_2_O‐CL. As temperature increases, Δ*G*_f_(*T*) for both the monocationic and bicationic compounds increase correspondingly, which implies a general decrease in thermal stability. Within this temperature range, Δ*G*_f_(*T*) of the vast majority of oxides are more negative than the corresponding nitrides in both bicationic and monocationic pairs. This is predominantly owing to the greatest scale of nitride metastability among all inorganic compounds.^[^ ^56^ ^]^ The kernel density distributions have also shown that the Δ*G*_f_(*T*) for bicationic compounds are generally more negative than those for the monocationic ones. It indicates that both the bicationic oxides and nitrides are statistically more stable than their monocationic counterparts.

Figure 2b compares the Δ*G*_f_(*T*) of nitrogen rich (N_rich_) and poor (N_poor_) nitride pairs in H_2_‐CL. A maximum temperature of 1200 K is considered because of the generally lower temperature required for H_2_‐CL experiment.^[^ ^25^ ^]^ The trend of Δ*G*_f_(*T*) change with respect to increasing temperature is consistent with oxide/nitride pairs. However unlike the clear tendency of Δ*G*_f, oxide_ < Δ*G*_f, nitride_ observed for the vast majority of the nitride/oxide pairs, the Δ*G*_f_ values for both the N_rich_ and N_poor_ metal nitrides are similar to each other. Statistically, 47.8% of the M–N_rich_/N_poor_ and 61.3% of the M_1_–M_2_–N_rich_/N_poor_ prefer the N_rich_ phase at 0 K ($\Delta G_{f,N_{\mathrm{rich}}}<\Delta G_{f,N_{\mathrm{poor}}}$), and these numbers reduce to 35.9% and 48.0% at 1200 K respectively. It suggests that the increasing temperature would have larger impact on destabilizing the N_rich_ nitride for both groups. Also since Rxn II (nitrogen fixation) in H_2_‐CL only involves the conversion between these two nitrides, the differences in the Δ*G*_f_ between these two phases would directly determine the energetics of this reaction.

#### 2.1.2 Gibbs Reaction Energies of Sub‐Reactions

**Figure 3:**
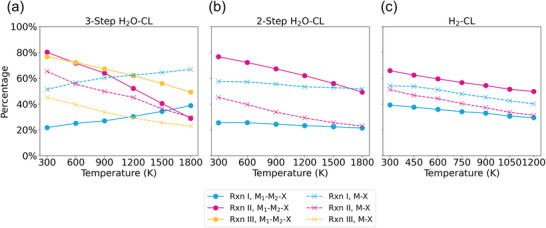
Percentages of redox pairs that possess spontaneous sub‐reaction (Δ*G*_r_ < 0) at different temperatures that can practically be reached in different CLAS processes: a) three‐step H_2_O‐CL, b) two‐step H_2_O‐CL, and c) H_2_‐CL. The solid lines correspond to CLs using bicationic compounds M_1_–M_2_–X as the active materials, whereas the dashed lines correspond to those using monocationic compounds M–X as the active materials. The blue, magenta, and orange colors are used to distinguish the sub‐reactions shown in Figure 1. Rxn II in the two‐step H_2_O‐CL (magenta lines) are identical to the Rxn III (orange lines) in the three‐step H_2_O‐CL, because they both correspond to the same chemical reaction of hydrolyzing the nitrides back to the oxides.

With the determinations of the Δ*G*_f_ values for different compounds to be used as the redox pairs, the Gibbs reaction energies (Δ*G*_r_) for each constituent chemical reactions can be subsequently calculated based on Equation (10). The proportions of the spontaneous chemical reactions being identified (i.e., those with Δ*G*_r_ < 0) are plotted in **Figure** **3** as a function of the reaction temperature, whereas more detailed mappings of Δ*G*_r_ for each redox pair are shown in Figures S1– S3, Supporting Information. It can be seen from Figure 3 that, most of the chemical reactions tend to proceed spontaneously at lower reaction temperatures, which is a desirable feature for the industrial development of ammonia synthesis with lower energy consumption. However, exceptions to this trend can be found for the reduction process that involves M_1_–M_2_–O and M–O in the three‐step H_2_O‐CL (Rxn I, Figure 3a), where the equilibria are shifted toward the product formations at the higher reaction temperatures. Practically, this implies that a significant temperature swing through different stages in three‐step H_2_O‐CL is necessary to enhance both the yield of NH_3_ and high turn‐over of the active materials.

Figure 3a shows that for Rxn I in three‐step H_2_O‐CL (reduction of oxides), less bicationic oxides M_1_–M_2_–O are likely to be spontaneously reduced to the corresponding metals compared to monocationic oxides M–O (the blue solid line is below the blue dashed line). However in subsequent Rxn II (nitridation, colored as magenta) and Rxn III (hydrolysis, colored as yellow), many more reactions driven by the bicationic redox pairs M_1_–M_2_–O/N are likely to proceed compared to those driven by the monocationic ones. Statistically, 21.8% and 51.4% of M_1_–M_2_–O and M–O can undergo spontaneous oxide reduction at 300 K, respectively. Although significantly more oxides can be reduced at a higher temperature of 1800 K, it remains easier to reduce the M–O (66.8%) compared to M_1_–M_2_–O (38.8%). On the other hand, 80.2% and 76.7% of the bicationic systems can undergo spontaneous nitridation and hydrolysis reactions at 300 K, which are higher than the percentages of the same chemical reactions involving the monocationic ones (65.3% and 45.1% respectively). This shows that the bicationic pairs M_1_–M_2_–O/N are clearly preferred in both two steps at low temperature. However, the numbers of spontaneous reactions decrease drastically with the rising temperature, especially in the nitridation process. At extremely high temperature of 1800 K, we see no advantage in the nitridation of bicationic nitrides M_1_–M_2_–N compared to the monocationic nitrides M–N, as indicated by 29.0% and 29.8% of all the reactions that may proceed spontaneously. The trends observed above can be mainly attributed to the change of Δ*G*_f_ for M_1_–M_2_–N and M–N in Figure 2a. As the stabilities of bicationic nitrides decrease, it then becomes more difficult for the nitridation reactions to proceed.

Figure 3b analyzes the percentages of sub‐reactions with negative Δ*G*_r_ in the two‐step H_2_O‐CL. In this process, the nitrogen fixation is carried out by purging the mixed gases of H_2_ and N_2_ simultaneously into the reactor to directly convert the oxide into the corresponding nitride (Rxn I). The ratios of Rxn I that can proceed spontaneously are nearly insensitive to the temperature variation, which remain at around 55% and 23% for M–O/N and M_1_–M_2_–O/N across the temperature range considered. This is because Rxn I in the two‐step H_2_O‐CL is obtained by combining the reduction and nitridation steps from three‐step H_2_O‐CL process together, which show opposite temperature‐dependent trends from each other.

For H_2_‐CL (Figure 3c), our analysis shows that M–N_rich_ can react with H_2_ easier to form NH_3_ (Rxn I, 54.1% for monocationic nitrides compared to 39.2% for bicationic ones at 300 K), whereas M_1_–M_2_–N_rich_ are slightly more stable (cf., Figure 2b), such that more nitrogen fixation reactions (Rxn II, 51.2% and 65.9% for mono‐ and bicationic nitrides at 300 K, respectively) occur spontaneously in this case. It shows that whilst M_1_–M_2_–N systems may have stronger propensity to capture the atmospheric N_2_ and form the nitrogen‐rich phase, it is harder to dissociate the metal–nitrogen bonds to form NH_3_ compared to the monocationic nitrides.

#### 2.1.3 Assessment of Limiting Energies and Reactions

In order to cross‐compare different CLAS processes that each consists of multiple sub‐reactions, thus facilitating the identification of the best redox pairs, we calculated the limiting energies (Δ*G*_r, lim_) for all loops carried out by either monocationic or bicationic redox pairs using Equation (12). We note that, a limiting reaction^[^ ^48^ ^]^ is the sub‐reaction in a specific CL process that contributes to the limiting energy (Δ*G*_r, lim_), in the temperature range considered. As such, Δ*G*_r, lim_ < 0 would imply that all steps within the reaction cycle will proceed spontaneously at given temperatures, and redox pairs that will contribute to negative Δ*G*_r, lim_ are highly desirable. Nevertheless, it should be noted that having negative Δ*G*_r, lim_ is a necessary but insufficient thermodynamic requirement for solid‐state CLAS to proceed. This is because the chemical reactivity can inevitably be affected by many other factors, such as reaction barrier heights, phase transition, dimensions (e.g., surface effects), and so on. The considerations of these influencing factors are beyond the scope of the present investigation.

**Figure 4:**
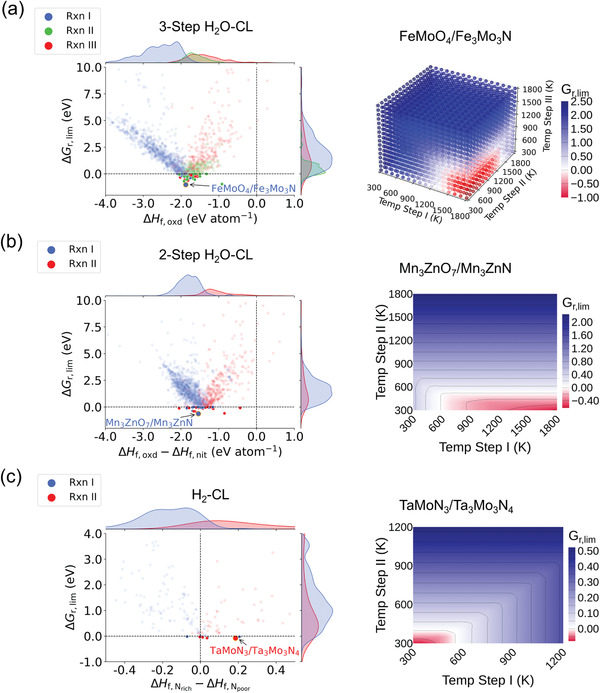
Volcano plots of the limiting energies using bicationic redox materials (M_1_–M_2_–X) in a) three‐step H_2_O‐CL, b) two‐step H_2_O‐CL, and c) H_2_‐CL. Δ*G*_r, lim_ for the candidates with the most negative values are also plotted for each CL (FeMoO_4_/Fe_3_Mo_3_N for three‐step H_2_O‐CL, Mn_3_ZnO_7_/Mn_3_ZnN for two‐step H_2_O‐CL, and TaMoN_3_/TaMoN_3_ for H_2_‐CL) with respect to the reaction temperatures in the looping processes. The limiting reactions are marked as blue, green, and red for Rxn I, II, and III in the three‐step H_2_O‐CL and as blue and red for Rxn I and Rxn II in the rest two‐step processes respectively (b and c).

In **Figure** **4**, we plot the Δ*G*_r, lim_ for CLs involving bicationic redox pairs with respect to either the formation enthalpies Δ*H*_f_ of the active oxides (Figure 4a), or the differences in the Δ*H*_f_ (i.e., ΔΔ*H*_f_ Figure 4b,c) between the redox pairs, in order to reveal how Δ*G*_r, lim_ for different sub‐reactions are fundamentally determined by Δ*H*_f_. (Similar plots for the monocationic systems can be found in Figure S4, Supporting Information.) This gives rise to the so‐called “volcano plots,” and the choice of presenting Δ*G*_r, lim_ with respect to either Δ*H*_f_ or ΔΔ*H*_f_ depends on whether both the reduced and oxidized forms of the redox pair are coexisting in all sub‐reactions. More detailed explanation on these choices can be found in Section S3 and Figure S5, Supporting Information.

Figure 4a shows the Δ*G*_r, lim_ of three‐step H_2_O‐CL against Δ*H*_f, oxd_ (i.e., the formation enthalpies for the bicationic oxides). Statistically, we found that the limiting reactions for more than 60% (936 out of 1526) of the three‐step H_2_O‐CL are coming from the reduction step (Rxn I), whereas only 351 and 239 CL processes are limited by the hydrolysis (Rxn III) and the nitridation (Rxn II), respectively. Similar statistical trend for the monocationic redox pairs can also be observed in Figure S4a, Supporting Information. This shows that, for the three‐step H_2_O‐CL, the reduction of metal oxides to metals is the most difficult part to proceed in the entire loop.

Focusing on redox pairs that are able to drive spontaneous three‐step H_2_O‐CL (i.e., those possess negative Δ*G*_r, lim_), Figure 4a shows that, the Δ*H*_f, oxd_ for these pairs predominantly fall into a narrow region between −1.33 and −2.09 eV atom^−1^. When the oxides are too stable (Δ*H*_f, oxd_ < −2.09 eV atom^−1^), they become very difficult to be reduced in Rxn I, resulting in the increase of Δ*G*_r, lim_ with Rxn I as the limiting reaction. On the other hand, when the Δ*H*_f, oxd_ is larger than −1.33 eV atom^−1^, Rxn II or III now becomes the limiting reaction. The scenario for Rxn II being the limiting reaction can be attributed to the correlated Δ*G*_f_ between oxides and nitrides seen in Figure 2. When the oxides are less stable, the stabilities of the complementary nitrides would also decrease in most cases, which makes their formations more difficult from directly reacting the metals with N_2_ and therefore leads to the Rxn II becoming the limiting reaction. Similarly, when both the oxides and nitrides have Δ*H*_f_ that are too large, the energy released from the conversion of nitride back to the oxide will not be sufficient to drive the endothermic reaction that converts H_2_O to NH_3_ (At 300 K, $\Delta G_{f,H_{2}O}=-2.37\text{eV}\;{\text{atom}}^{-1}$ and $\Delta G_{{f,\mathrm{NH}}_{3}}=-0.17\text{eV}\;{\text{atom}}^{-1}$.^[^ ^57^ ^]^), which can also turn the hydrolysis reaction (Rxn III) into the limiting reaction.

Statistically, 97 three‐step H_2_O‐CL that are driven by the bicationic redox pairs could proceed spontaneously, which are 26% more than those driven by the monocationic ones. Among all viable candidates, FeMoO_4_/Fe_3_Mo_3_N gives the lowest Δ*G*_r, lim_ of −1.01 eV with Rxn I as the limiting reaction. To illustrate how this limiting reaction and the corresponding reaction free energy are determined, the right hand side of Figure 4a shows a plot of Δ*G*_r, lim_ as a function of the three sub‐reaction temperatures. It is immediately clear that the lowest Δ*G*_r, lim_ is reached when Rxn I is set at the highest reaction temperature of 1800 K while the temperatures for Rxn II and III are fixed at the lowest 300 K. The high (low) temperatures required for Rxn I (II/III) revealed in Figure 4a are also consistent with the trends observed in the Figure 3a for three‐step H_2_O‐CL. Moreover, it is also seen from the temperature‐dependency of Δ*G*_r, lim_ that there exist a range of temperatures in which Δ*G*_r, lim_ are negative. For example, the temperature for the oxide reduction (Rxn I) may be further lowered to 1400 K, at which the reaction can still proceed spontaneously, albeit with potentially slower reaction kinetics. It shows that this information is particularly useful to guide the optimization of the reaction conditions in a CL process.

Further examinations on the chemical constituents for all 97 lead candidates of the M_1_–M_2_–O/N redox pairs reveal many of them contain one or both cations of which corresponding monocationic counterparts (M–O/N) are not viable candidates, such as MgCoO_3_/MgCoN, Ca_2_Ag_2_O_5_/CaAgN, ZnCrO_3_/ZnCrN_2_, CoWO_4_/Co_3_W_3_N, etc. This further demonstrates the existence of new chemical bonding interactions in compounds with complex compositions plays a significant role in changing materials' thermodynamic stabilities, enabling them to become promising active materials that do no exist in the chemical space of simpler compounds.

The left hand side of Figure 4b shows the volcano plot for the two‐step H_2_O‐CL constructed for the bicationic redox pairs. It shows that the Δ*G*_r, lim_ are most negative when the oxides are ≈1.5 eV atom^−1^ enthalpically more stable that the corresponding nitrides. Statistically, the numbers of viable redox pairs for the two‐step process are 44 and 54 for M_1_–M_2_–O/N and M–O/N (see also Figure S4b, Supporting Information), which are less than those for the three‐step cycle. Notably the number of bicationic redox pairs has been halved indicating that there is no significant benefit in using bicationic compounds to drive the two‐step CL processes. Meanwhile, the Δ*G*_r, lim_ values for many redox pairs that are negative in the three‐step cycle have increased significantly in the two‐step process. For instance, Δ*G*_r, lim_ using KSe_2_O/K_2_Se_4_N as the active materials increases from −0.96 eV atom^−1^ in the three‐step cycle to 0.99 eV atom^−1^ calculated for the two‐step one. It shows thermodynamically, the direct conversion of oxides to their corresponding nitrides without the formation of the metallic intermediates could be challenging to proceed. Within these candidates in the two‐step process, Mn_3_ZnO_7_/Mn_3_ZnN has the most negative Δ*G*_r, lim_ of −0.62 eV atom^−1^, where its temperature dependence is shown in the contour plot in Figure 4b (right panel). The applicable temperatures of Rxn I have a particularly wide range from 600 to 1800 K to deliver Δ*G*_r_ < 0. In contrast, Rxn II is exothermic only when the hydrolysis temperature is below 500 K.

Finally, Figure 4c demonstrates that the reaction spontaneity for the H_2_‐CL is also largely dependent upon the differences in the formation enthalpies between the N_rich_ and N_poor_ phases of the metal nitrides. Unlike the trends observed in the previous two CL processes, the two nitride phases should have comparable formation enthalpies in order to lower the overall Δ*G*_r_. This is because H_2_O, as being thermodynamically very stable, is not involved in this particular CL process. When the N_rich_ phase is more stable than the N_poor_ phase, that is, $\Delta H_{f,N_{\mathrm{rich}}}-\Delta H_{f,N_{\mathrm{poor}}}<0$, the main energy barrier for the entire CL process is contributed by the Rxn I. This implies the difficulty of releasing chemically bonded nitrogen from the crystalline lattice to combine with H_2_ in the environment to form NH_3_. In contrast, nitrogen fixation in Rxn II will become difficult to proceed when the N_poor_ compound is energetically more stable as indicated by $\Delta H_{f,N_{\mathrm{rich}}}-\Delta H_{f,N_{\mathrm{poor}}}>0$. More specifically, only nine bicationic nitride pairs are found to possess negative Δ*G*_r, lim_ whereas for monocationic nitride pairs, this number is 28 (Figure S4c, Supporting Information). This is mainly due to the fact that the number of bicationic nitrides that can be extracted from the MP database is only one third of the monocationic nitrides.

Overall, our screening results show that the thermodynamic driving force of H_2_‐CL is much lower than the other two CL processes with Δ*G*_r, lim_ < −0.1 eV for all viable monocationic and bicationic redox pairs. This leads to a much narrower temperature range in which the CL process might proceed spontaneously, as demonstrated by the temperature‐dependent contour plots for TaMoN_3_/TaMoN_3_ (right panel in Figure 4c) and CaN_6_/Ca_3_N_2_ (right panel in Figure S4c, Supporting Information). The low reaction temperatures could potentially lead to sluggish kinetics, therefore further hinder the yield of NH_3_.

### 2.2 Diversifying the Chemical Space to Discover Redox Pairs for MH‐CL

As previously shown in Table 1, the MP database does not contain enough number of compounds that will enable us to achieve a thorough screening of redox pairs suitable for MH‐CL. To overcome this problem, we applied the strategy using elemental substitution of the existing materials in the MP database, in order to expand the chemical space.

**Figure 5:**
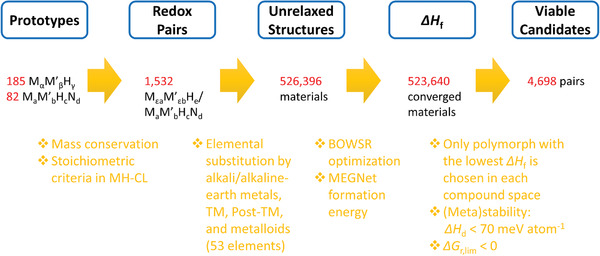
Workflow for diversifying the chemical space of metal hydride/nitride‐hydrides to discover suitable redox pairs for MH‐CL.

The workflow is illustrated in **Figure** **5**. We first constructed 1532 prototypical redox pairs from 185/82 unique bicationic hydrides/nitride‐hydrides extracted from the MP database. Then the cations in the prototypical bicationic redox pairs were both replaced by 53 different chemical elements consisting of alkali/alkaline‐earth metals, transition metals (TM), post‐transition metals (post‐TM), and metalloids, that are prior to the element Po. Such a strategy provided us with more than half a million structures to be screened. However, to obtain their Δ*H*_f_ values requires the crystal structures to be first optimized with first‐principles calculations, which is computationally unfeasible for a study in this scale. Instead, the Bayesian optimization with symmetry relaxation (BOWSR)^[^ ^58^ ^]^ algorithm was used that allowed us to optimize the crystal structures of the hypothetical compounds at a negligible cost. Then, Δ*H*_f_ of these optimized structures were predicted by a graph network (MEGNet)^[^ ^59^ ^]^ trained on the DFT formation energies for compounds existing in the MP database. The benchmarking results and parameters used for predicting the Δ*H*_f_ of the hypothetical materials in this work are discussed in Figure S6, Supporting Information. With this, Δ*H*_f_ for 523 640 structures were successfully obtained.

Three energy metrics were then applied to hierarchically filter out the viable candidates for the subsequent screening of the reaction thermodynamics. First, only the most stable polymorph with the lowest Δ*H*_f_ was retained for a given compound. Second, the decomposition enthalpy^[^ ^60^, ^61^ ^]^ (Δ*H*_d_) was calculated for the lowest energy polymorphs and those with Δ*H*_d_ < 70 meV atom^−1^ were chosen for the subsequent reaction thermodynamic screenings.^[^ ^56^, ^62^ ^]^ The threshold set for Δ*H*_d_ is to ensure the hypothetical bicationic hydrides and nitride‐hydrides to be (meta)stable in competing with other compounds in the corresponding chemical space. From the consideration of practicality, it indicates that the filtered candidates are most likely to be experimentally synthesizable.^[^ ^63^, ^64^ ^]^ Finally, similar to the proceeding analysis of H_2_O‐CL and H_2_‐CL, Δ*G*_r, lim_ was calculated to assess the cyclic spontaneity of the MH‐CL under elevated temperatures.

**Figure 6:**
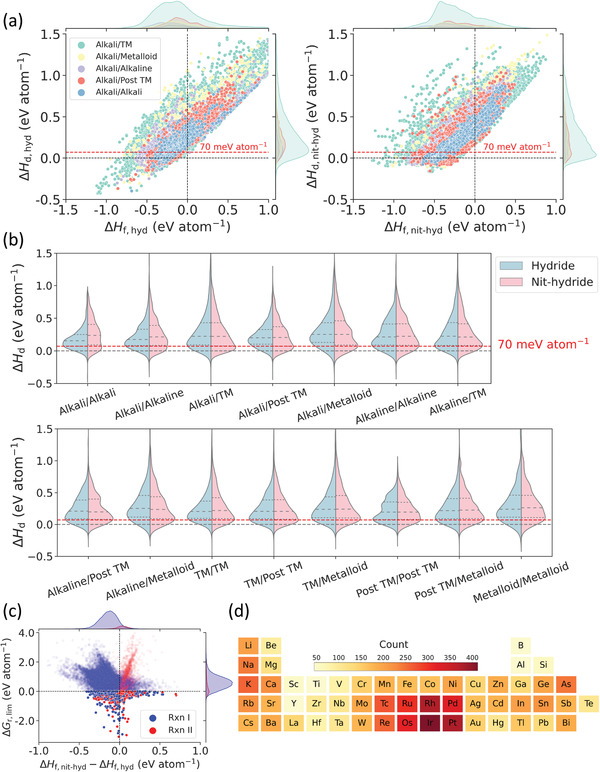
Analysis of the chemical space consisting the hypothetical hydrides/nitride‐hydrides redox pairs that may be used in MH‐CL. a) Correlations between Δ*H*_d_ and Δ*H*_f_ for a subset of bicationic hydrides (left panel) and nitride‐hydrides (right panel) containing alkali metals. Groups with different second cations are colored in green, yellow, purple, red, and blue for transition metals (TM), metalloids, alkaline‐earth metals, post‐transition metals (post‐TM), and alkali metals respectively. b) Violin plots of the distribution of decomposition energies as categorized by cation groups. Hydrides and nitride‐hydrides are separately colored in light blue and pink, respectively. The dashed lines in each violin mark the quartiles of the distribution. c) Volcano plot of Δ*G*_r, lim_ for (meta)stable redox pairs where limiting reactions are colored in blue and red to specify Rxn I and II respectively. Highlighted dots are the viable candidates with negative Δ*G*_r, lim_. d) Frequency of cation appearance in the viable redox pairs with Δ*G*_r, lim_ < 0.

To illustrate how Δ*H*_f_ and Δ*H*_d_ affect each other, **Figure** **6**a shows plots of Δ*H*_d_ against Δ*H*_f_ for a subset of bicationic metal M_1_–M_2_–H (left panel) and M_1_–M_2_–N–H (right panel), respectively, with at least one cation being alkali metal. Most of the compounds screened here contain at least one TM element as cation, which is because the majority of cation candidates used for the initial model generation are TMs. While Δ*H*_f_ depicts the energy difference between the compound and its constituent elements only, Δ*H*_d_, by definition, represents the relative stability of a specific phase to its competing phases. Therefore, Δ*H*_d_ is often considered as a much more rigorous criterion for judging the synthesizability of a hypothetical material.^[^ ^65^ ^]^ 70% of M_1_–M_2_–H and 79% of M_1_–M_2_–N–H are with Δ*H*_f_ < 0. In contrast, only around 7% of M_1_–M_2_–H and 9% of M_1_–M_2_–N–H are found to possess Δ*H*_d_ < 0. Nevertheless, it was found that the metastable phases of many materials (as characterized by a positive Δ*H*_d_) could also be synthesized.^[^ ^66^, ^67^ ^]^ As such, it is necessary to slightly relax the threshold of the Δ*H*_d_ filter. Here, by setting Δ*H*_d_ < 70 meV atom^−1^, 3,914 M_1_–M_2_–H, and 5,187 M_1_–M_2_–N–H are retained.

In Figure 6b, the distributions of Δ*H*_d_ for bicationic compounds with different combinations of metal cations are summarized, which further demonstrates that around 20% (meta)stable structures for each subgroup of compounds are included with the applied filtering criterion based on Δ*H*_d_. It is also clear from Figure 6b that, the nitride‐hydrides are generally more stable than the pure hydrides, which would significantly influence the reaction energies that convert between these two types of compounds.

Focusing more on the chemical trends of the stable compounds (Figure S7, Supporting Information), it is found that the combination of alkali metal and post‐TM has the highest probability (24.9%) of forming (meta)stable M_1_–M_2_–N–H. This is closely followed by a group of materials composed by alkali metal and TM (23.9%), of which the experimental success on MH‐CL has been recently reported.^[^ ^68^ ^]^ For M_1_–M_2_–H, 22.0% of those that contain one alkaline‐earth metal and one TM are (meta)stable, which is the most preferred cation combination that involves a TM. Although M_1_–M_2_–N–H are more stable than the M_1_–M_2_–H, we do observe exception where intermixing two alkali metals gives more stable hydrides (24.9%) in comparison to their nitride‐hydride counterparts (22.2%).

Moving on to examine how the phase stabilities of bicationic metal hydrides/nitride‐hydrides affect the reaction thermodynamics of MH‐CL, Figure 6c demonstrates the volcano plot that correlates Δ*G*_r, lim_ with Δ*H*_f, nit‐hyd_ − Δ*H*_f, hyd_ of (meta)stable M_1_–M_2_–N–H/M_1_–M_2_–H that may be used for MH‐CL. 90.5% of the limiting reactions are found to be the hydrogenation of M_1_–M_2_–N–H (Rxn I) to form NH_3_. It is due to the higher phase stability of nitride‐hydrides that makes the formation of the pure hydrides become less favored. Amongst 45,467 hypothetical redox pairs that have been constructed, only ≈10% of them have negative Δ*G*_r, lim_ within the formation energy window of Δ*H*_f, nit‐hyd_ − Δ*H*_f, hyd_ ∈ [ − 0.40, 0.70] eV atom^−1^. It shows that spontaneous conversion through MH‐CL is only possible when the metal hydrides and their corresponding nitride‐hydrides have comparable formation enthalpies.

Figure 6d visualizes the occurrence of cations in these viable redox pairs to reveal the preferred chemical compositions of redox pairs for MH‐CL. The most frequently occurring cations are from the TMs, especially precious metals Ir, Pt, Rh, Pd, Os, and Ru. Furthermore, many systems containing alkali (K, Na, Li, and Rb) and alkaline‐earth metals (Ca, Sr, and Ba) also occur frequently. Examining what cation combinations could lead to negative Δ*G*_r, lim_, similar patterns can also be observed in Figure S8a, Supporting Information. After excluding precious metals and toxic/radioactive elements (e.g., As, Hg, Tc, etc.), more cost‐effective and sustainable bicationic pairs can be found in Figure S8b, Supporting Information. In these candidates, experimental successes of redox materials involving Ni/Mn/Mo and alkali/alkaline‐earth metals have been reported in recent studies.^[^ ^31^, ^68^ ^]^ More significantly, new cation combinations containing In–Mo, Fe–Mn, and In–W (in the families of TM/post‐TM and TM/TM) have also shown promising potentials to be applied for MH‐CL, with suitable phase stabilities and limiting reaction free energies.

### 2.3 Exploring the Cooperative Effects in Bicationic Redox Pairs Using Three‐Step H_2_O‐CL as an Example

So far, we have successfully screened the reaction thermodynamics for the four chosen CL processes using both monocationic and bicationic redox pairs, from which the viable pairs for each process have been identified. However, it does not provide us with the information to judge whether the unique interactions between two heterocations in bicationic redox pairs M_1_–M_2_–X could indeed enhance the performance of chemical looping compared to the two corresponding monocationic counterparts, that is, M_1_–X and M_2_–X.

Hereby, we attempt to shed light on this question by analyzing the differences in Δ*G*_r, lim_ for chemical looping processes that are enabled by M_1_–M_2_–X to those facilitated by the corresponding monocationic counterparts M_i_–X (i = 1, 2). For this, we define a measuring metric $\lambda =\frac{\Delta \Delta G_{1}^{\lim}+\Delta \Delta G_{2}^{\lim}}{|\Delta \Delta G_{1}^{\lim}-\Delta \Delta G_{2}^{\lim}|}=\frac{\Delta \Delta G_{1}^{\lim}+\Delta \Delta G_{2}^{\lim}}{|\Delta G_{M_{1}X}^{\lim}-\Delta G_{M_{2}X}^{\lim}|}$ where $\Delta \Delta G_{i}^{\lim}=\Delta G_{M_{1}M_{2}X}^{\lim}-\Delta G_{M_{i}X}^{\lim}$ is the difference in the limiting reaction free energies between CLs that are driven by M_1_–M_2_–X and M_i_–X. With this definition, we categorize the thermodynamics of three‐step H_2_O‐CLs that are driven by the bicationic redox pairs as being: 1) Cooperatively enhanced (λ < −1, occurs when $\Delta G_{M_{1}M_{2}X}^{\lim}<\min\left\{\Delta G_{M_{1}X}^{\lim},\Delta G_{M_{2}X}^{\lim}\right\}$), which means the intermixing of two different cations in a single bicationic compound creates new bonding states that are beneficial in facilitating the NH_3_ formations in three‐step H_2_O‐CL. 2) Additive effects (λ ∈ [ − 1, 1]), meaning that from the thermodynamic perspective, the performances of the M_1_–M_2_–X are comparable to the M_i_–X (i = 1,2). And 3) anti‐cooperative (λ > 1, occurs when $\Delta G_{M_{1}M_{2}X}^{\lim}>\max\left\{\Delta G_{M_{1}X}^{\lim},\Delta G_{M_{2}X}^{\lim}\right\}$), which corresponds to a situation where the looping performances of using a bicationic compound as the active material are worsened compared to using either one of the two corresponding monocationic redox materials. A more detailed explanation of the correlation between λ and the limiting energies of M_1_–M_2_–X and M_i_–X can be found in Figure S9, Supporting Information.

**Figure 7:**
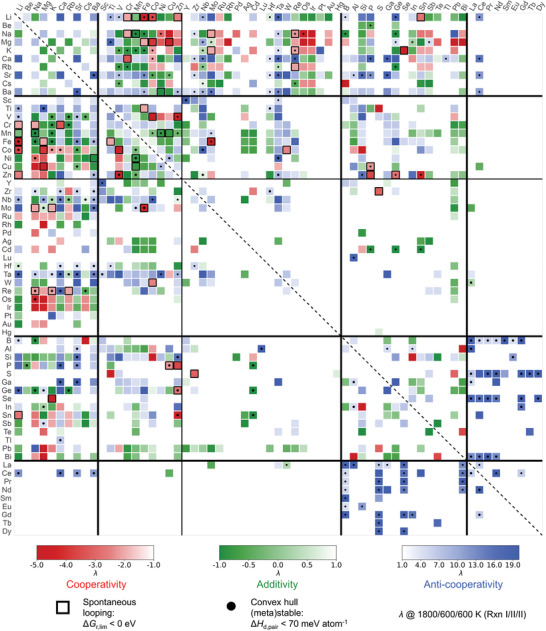
Heatmap of λ between bicationic and monocationic redox pairs for three‐step H_2_O‐CL. Blue color indicates both monocationic pairs are more favorable than the bicationic ones (λ > 1). Green color indicates the bicationic pairs are merely showing additive effect from the constituent cations (−1 < λ < 1). Most importantly, red color highlights the bicationic pairs with cooperative enhancement (λ < −1). The elements are grouped by their positions in the periodic table, that is, s‐block (alkali/alkaline‐earth metals), d‐block (transition metals), p‐block (post‐transition metals, metalloids, and nonmetals), and 4f‐block. 3d‐transition metals are further separated because of their highest rate of cooperative enhancement, convex hull (meta)stability, and looping spontaneity. Viable pairs with negative limiting energies are labeled by black squares. (Meta)stable bicationic pairs of which redox materials have decomposition energies both smaller than 70 eV atom^−1^ are marked by black dots. An interactive version of this map can be found in Supporting Information.

Here, we focus our analysis of λ using three‐step H_2_O‐CL as an example, due to the long‐lasting interest of applying this looping mechanism in the research community of ammonia synthesis. The results are presented in the matrix plot shown in **Figure** **7**. The matrix plot is symmetrical about its diagonal because the cations M_1_ and M_2_ in the chemical formulas are treated equivalently. Each block of the matrix plot is color‐coded according to the λ calculated for that particular combination of cations.

Overall, as revealed in the heat map, most bicationic redox pairs (46.3%) are showing an anti‐cooperative effect (λ > 1). It implies that for the majority of the three‐step H_2_O‐CL, using the bicationic redox pair as the active material makes the limiting reaction less likely to proceed spontaneously, compared to the case where either one of the corresponding monocationic redox pairs is used. It also indicates that compounds with more complex chemical compositions do not necessarily make them better active materials for thermal ammonia synthesis, which may also explain why most of the prior studies were focusing on the monocationic systems. 36.9% cases show additive effect (−1 ⩽ λ ⩽ 1), which means performance‐wise, using the bicationic active materials is equivalent to the usage of a simple mixture of the two monocationic compounds. It also indicates that mixing two cations in a single compound did not create different chemical bondings that will fundamentally change the thermodynamic behaviors of the bicationic compounds In stark contrast, only 93 (16.8%) CLs show cooperatively enhanced spontaneity for the limiting reaction when the bicationic redox pair is used as indicated by λ < −1. This occurs for a few groups of bicationic compounds scattered across the map shown in Figure 7.

To enable a better judgement on the deviation of $\Delta G_{M_{1}M_{2}X}^{\lim}$ from the Δ*G* ^lim^ of the monocationic counterparts, we also map out the limiting energy differences defined as $\Delta \Delta G_{r,\lim}=\max\left\{\Delta \Delta G_{1}^{\lim},\Delta \Delta G_{2}^{\lim}\right\}$ in Figure S10, Supporting Information. A graphical illustration behind the physical meaning of ΔΔ*G*_r, lim_ can be found in Figure S11, Supporting Information. Compared to the categorical λ index, ΔΔ*G*_r, lim_ provides a more intuitive representation regarding the distance between the bicationic pair and the thermodynamically more viable monocationic pair on the energy scale. Amongst the pairs that are showing the cooperative enhancement effect, the majority of them are compounds that contain a combination of alkali/alkaline‐earth metals and TM/post‐TM/metalloids. Notably, KSe_2_O/K_2_Se_4_N has the most significant improvement in limiting energy with ΔΔ*G*_r, lim_ = −2.2 eV. Many M_1_–M_2_–O/N containing Mg have also shown drastic improvement in limiting energy where Mg_2_Cu_2_O_5_/MgCuN has the most negative ΔΔ*G*_r, lim_ of −1.3 eV amongst all the bicationic systems.

Moreover, the combination between alkali/alkaline‐earth metals and TM has the most redox materials that exhibit cooperative enhancement (Figure S12, Supporting Information). Further examination on the other energetic metrics of these redox pairs reveals that a subset of M_1_–M_2_–O/N comprising 3d–TM tend to have low Δ*H*_d_ (marked with black dots in Figures 7 and S10, Supporting Information) and negative Δ*G*_r, lim_ (highlighted with black squares), which further increases their feasibility in experimental practice. LiCoO_2_/LiCoN and Li_3_FeO_4_/Li_3_FeN_2_ are of particular interest since they fulfill the three energy metrics simultaneously. This implies that by coupling Co/Fe with Li to facilitate three‐step H_2_O‐CL, not only the transformation between the M_1_–M_2_–N and M_1_–M_2_–O is predicted to be energetically preferred in competing with other phases, the overall looping thermodynamics is also synergistically enhanced compared to their monocationic counterparts.

Apart from alkali/alkaline‐earth and TM, several pairs with different combinations of elements have also shown cooperative effect together with their high convex hull stabilities and looping spontaneity, including FeMoO_4_/Fe_3_Mo_3_N, ZnGeO_3_/ZnGeN_2_, V_2_Zn_4_O_9_/VZn_2_N_3_, Cu_2_P_2_O_7_/CuPN_2_, NaReO_3_/NaReN_2_, and KReO_3_/KReN_2_. In consideration of the high reactivity of alkali/alkaline‐earth metals, the high volatility of P, and the scarcity of Re, the two redox pairs (FeMoO_4_/Fe_3_Mo_3_N, ΔΔ*G*_r, lim_ = −0.469 eV, and ZnGeO_3_/ZnGeN_2_, ΔΔ*G*_r, lim_ = −0.703 eV) comprising TM and metalloid elements may be more suitable to be applied in NH_3_ synthesis at a higher temperature. In fact, the experimental evidences of nitrogen reduction reaction have been reported on both FeMoO_4_^[^ ^69^, ^70^, ^71^ ^]^ and Fe_3_Mo_3_N,^[^ ^72^ ^]^ which further increases the practicability of this pair.

## 3 Conclusions

Chemical looping is a promising alternative to the traditional Haber–Bosch process for ammonia synthesis, in which the thermal energy input may be sourced from renewable energies, such as solar‐thermal, geothermal, and waste heats from thermal power plants. Successful delivery of this technology is heavily relying on the discovery of new active redox materials that can facilitate these looped chemical reactions with high efficiency.

In this work, we applied high‐throughput screening to assess the potential applicability of bicationic inorganic compounds as the active redox materials for ammonia synthesis in four different CL processes. We further compared their performances to the corresponding monocationic compounds in order to reveal how the reaction thermodynamics may be affected by the increase of the chemical complexity of the redox materials. With the usage of a machine‐learned Gibbs free energy descriptor, the reaction free energies for H_2_O‐CLs and H_2_‐CL that involves 1528 M_1_–M_2_–O/N and 173 M_1_–M_2_–N_rich_/N_poor_ active materials were systematically screened. From the volcano relationships that correlate the limiting reaction free energies with the formation enthalpies of the active redox materials, we discovered that CLs driven by the bicationic active materials tend to be thermodynamically limited by a different sub‐reaction. More importantly, the number of viable bicationic redox pairs and their corresponding chemical diversity increased significantly for the three‐step H_2_O‐CL, whereby 97 pairs have been predicted to be capable of driving the chemical loop spontaneously with novel cations that are absent in the workable monocationic counterparts.

Moreover, to overcome the scarcity of redox pairs extracted from the MP database that may be suitable for driving the MH‐CL process, we combined the strategies of cation substitutions and data‐driven ultrafast structural optimization, to significantly expand the chemical space of bicationic hydrides and nitride‐hydrides. This enables us to further identify 4698 candidates that may potentially be able to drive the MH‐CL process. In particular, we discovered that bicationic system with alkali/alkaline‐earth metals and TM/post‐TM elements are particularly promising candidates.

Last, by analyzing the differences in the Δ*G*_r, lim_ between M_1_–M_2_–O/N and the complementary monocationic redox pairs in three‐step H_2_O‐CL, we are able to further discover which bicationic redox materials can significantly lower the limiting reaction free energies compare to the cases where a simple mixture of two monocationic redox materials is used as the active materials. Among these candidates, LiCoO_2_/LiCoN, Li_3_FeO_4_/Li_3_FeN_2_, FeMoO_4_/Fe_3_Mo_3_N, and ZnGeO_3_/ZnGeN_2_ are pairs that show significant “cooperative enhancement” effect while possess low decomposition energies and ideal looping spontaneity.

Notably, many other important factors that could affect the chemical reaction outcomes of ammonia synthesis have not been considered in the current work, such as pressure, reaction kinetics, as well as the possible existence of the competing reactions. Most of the chemical reactions between solid materials and gaseous environment occur on surfaces, on which the reaction thermochemistries can differ considerably compared to the usage of the active materials in bulk form. Such effect is also not accounted for in the current theoretical model. For the lead candidates identified from our screening study, surface models of different crystallographic planes can be built, on which detailed reaction pathway calculations for gas dissociation (for N_2_, H_2_, or H_2_O) and ammonia formation can be performed using DFT. From here, the reaction energy barriers can be extracted for kinetic Monte Carlo simulations to model the reaction kinetics and yields.^[^ ^73^, ^74^, ^75^ ^]^ This will inevitably come at much higher computational costs than those employed in our present study. In light of this, we also note that some recent works have proposed new machine‐learning approaches to expedite the prediction of reaction pathways,^[^ ^76^, ^77^ ^]^ bring promise to study the catalytic reactions that involve more complex systems. How the cooperative enhancements in bicationic redox materials are affected by their unique electronic structures will also need to be further examined with detailed DFT calculations. Finally, to compete the cycle of theory‐driven material designs, the synthesizability of the lead bicationic compounds and their chemical looping performances under realistic experimental conditions should also be investigated. These will be the subjects of our future studies.

## 4 Experimental Section

### Expanding Redox Materials from Monocationic to Bicationic Compounds:

In the conventional chemical looping processes that involved two sub‐reactions (Figure 1a,c,d), completion of the chemical loop was accompanied with the yield of the product (NH_3_ in this case) and reoxidation of the reduced material back to its original state.^[^ ^78^, ^79^ ^]^ For instance, in a two‐step H_2_O‐CL, considering a generic binary metal oxide M_*a*_O_*b*_ and nitride M_*c*_N_*d*_ pair, the chemical cycle can be represented by the following two equilibrium reactions (given as per mole of NH_3_ synthesized per cycle):

I. Nitrogen fixation
(1) $$\frac{c}{ad}M_{a}O_{b}+\frac{bc}{ad}H_{2}+\frac{1}{2}N_{2}\to \frac{1}{d}M_{c}N_{d}+\frac{bc}{ad}H_{2}O$$
II. Hydrolysis
(2) $$\frac{1}{d}M_{c}N_{d}+\frac{bc}{ad}H_{2}O\to \frac{c}{ad}M_{a}O_{b}+{\text{NH}}_{3}+\left(\frac{bc}{ad}-\frac{3}{2}\right)H_{2}$$

Here, the metal oxide was first placed in the environment of mixed N_2_ and H_2_ where it can be reduced and nitridated to form the corresponding nitride. Then the nitride was put in contact with water vapor to yield ammonia and regenerated the oxide thus completing the loop. Analogously, for using a generic ternary pair $M_{\alpha }M_{\beta }^{\prime }O_{\gamma }$/$M_{\varepsilon \alpha }M_{\varepsilon \beta }^{\prime }N_{\theta }$ as the active materials, the same chemical reactions can be rewritten as

I. Nitrogen fixation
(3) $$\frac{\varepsilon }{\theta }M_{\alpha }M_{\beta }^{\prime }O_{\gamma }+\frac{\varepsilon \gamma }{\theta }H_{2}+\frac{1}{2}N_{2}\to \frac{1}{\theta }M_{\varepsilon \alpha }M_{\varepsilon \beta }^{\prime }N_{\theta }+\frac{\varepsilon \gamma }{\theta }H_{2}O$$
II. Hydrolysis
(4) $$\frac{1}{\theta }M_{\varepsilon \alpha }M_{\varepsilon \beta }^{\prime }N_{\theta }+\frac{\varepsilon \gamma }{\theta }H_{2}O\to \frac{\varepsilon }{\theta }M_{\alpha }M_{\beta }^{\prime }O_{\gamma }+{\text{NH}}_{3}+\left(\frac{\varepsilon \gamma }{\theta }-\frac{3}{2}\right)H_{2}$$

Here, the possible binary oxide intermediates were excluded in order to purely focus on the ternary transformation. An important scale factor ε > 0 was introduced, which chemically implied that the relative molar ratio between M and M′ is preserved in Equation (4) to enforce the mass conservation of cations. It was also worth emphasizing that for both the binary and the ternary reactions, hydrogen should appear as the reaction product rather than the reactants in the hydrolysis process (Equations (2) and (4)), and such condition can be enforced by a non‐negativity constraint on the coefficients $\frac{bc}{ad}-\frac{3}{2}$ and $\frac{\varepsilon \gamma }{\theta }-\frac{3}{2}$.

The only feature that distinguished the three‐step H_2_O‐CL from the 2‐step ones was the nitrogen fixation step was further split into two independent processes carrying out oxide reduction and metal nitridation separately:

I. Reduction
(5) $$\frac{\varepsilon }{\theta }M_{\alpha }M_{\beta }^{\prime }O_{\gamma }+\frac{\varepsilon \gamma }{\theta }H_{2}\to \frac{\varepsilon \alpha }{\theta }M+\frac{\varepsilon \beta }{\theta }M^{\prime }+\frac{\varepsilon \gamma }{\theta }H_{2}O$$
II. Nitridation
(6) $$\frac{\varepsilon \alpha }{\theta }M+\frac{\varepsilon \beta }{\theta }M^{\prime }+\frac{1}{2}N_{2}\to \frac{1}{\theta }M_{\varepsilon \alpha }M_{\varepsilon \beta }^{\prime }N_{\theta }$$

It was clear that Equation (3) can be obtained by summing Equations (5) and (6) together. Here the products were constrained in the reduction step to be two pure metals to avoid the complexity of binary metal alloy formation with different possible stoichiometries.^[^ ^80^ ^]^ As a result, the subsequent nitridation step was the formation reaction of ternary nitride from its constituent elements. The rest of the chemical equations for both bicationic and monocationic redox pairs applied in studied CLs can be found in Section S1, Supporting Information.

### Machine Learning Approaches for Fast Free Energy Calculations: Gibbs Free Energy Descriptor:

The Gibbs free energy was the fundamental variable that governed the thermodynamics of CLAS reactions, which enables to determine the likelihood for a given chemical reaction to proceed under a specific thermodynamic condition. However the experimental Gibbs free energies were only available for a small fraction of known inorganic solids.^[^ ^57^ ^]^ Conventional computational approach for determining the temperature‐dependent Gibbs free energies for solids involved calculating the phonon density‐of‐states for each material, which will not be feasible for conducting high‐throughput material screenings.^[^ ^81^ ^]^ To tackle this problem, recently, a new regression relationship that related the temperature‐dependent Gibbs free energies with basic atomic properties in a solid was established via machine‐learning,^[^ ^49^ ^]^ enabling the ultrafast estimation of the Gibbs free energy. In its essence, the temperature‐dependent Gibbs free energies can be determined from the following equations

(7) $$\Delta G_{f}\left(0\;K\right)=\Delta H_{f}\left(0\;K\right)$$

(8) $$\Delta G_{f}\left(T>0\;K\right)=\Delta H_{f}\left(0\;K\right)+G^{\delta }\left(T\right)-\sum_{i}^{N}\alpha_{i}G_{i}\left(T\right)$$

(9) $$\begin{array}{c} \begin{array}{c} G^{\delta }\left(T\right)=\left(-2.48\times 10^{-4}\ln\left(V\right)-8.94\times 10^{-5}{\text{mV}}^{-1}\right)T+0.181\times \ln\left(T\right)-0.882 \end{array} \end{array}$$

where Δ*H*_f_ is the formation enthalpy at 0 K, *G* ^δ^ the Gibbs energy descriptor that depends on the atomic volume *V*, the reduced atomic mass *m*, and the temperature *T*. α_i_ is the stoichiometric weight of the *i*th element in the compound and *G*_i_ the corresponding Gibbs free energy obtained from tabulated experimental or calculated results.^[^ ^49^ ^]^ In this work, the crystal structures and Δ*H*_f_ of inorganic solid compounds were retrieved from the MP^[^ ^50^ ^]^. It was worth emphasizing that only the polymorph with the lowest Δ*H*_f_ was considered in the calculations, where detailed descriptions of the workflow to retrieve and filter out data can be found in Section S2, Supporting Information. The algorithm used for computing the Δ*G*_f_(*T*) for all solids was written based on the GibbsComputedStructureEntry module in the pymatgen package,^[^ ^82^ ^]^ whereas the thermodynamic data of gaseous species (H_2_O, NH_3_) were extracted from the NIST‐JANAF tables of experimental thermochemical data. Benchmark of this physical descriptor with DFT calculations can be found in Section S4.1, Supporting Information.

### Machine Learning Approaches for Fast Free Energy Calculations: Structure Optimization and Formation Energy Prediction:

In order to further extend the searching domain of possible candidates, hypothetical cation‐substituted hydrides and nitride‐hydrides were constructed for MH‐CL and fast optimized by a recently‐released BOWSR.^[^ ^58^ ^]^ This algorithm utilized a surrogate model to determine the optimal lattice parameters and atomic coordinates. MEGNet ^[^ ^59^ ^]^ was subsequently used to predict the formation energies (Δ*H*_f_) of the optimized crystals, in which this machine learning model was trained on a large amount of DFT‐computed data from MP and had managed to achieve very low cross‐validated mean absolute error (MAE) of 26 meV atom^−1^ in a wide range of materials. The benchmarking results (Figure S6, Supporting Information) have shown that, by coupling BOWSR and MEGNet, this workflow was capable of producing reasonably accurate Δ*H*_f_ as compared to the expensive DFT‐computed energies. The MAEs calculated on the M_1_–M_2_–H and M_1_–M_2_–N–H datasets were both falling below the reported average value, which made it a reliable approach for energy predictions while greatly boosting the screening efficiency. Benchmark of this machine learning workflow with DFT calculations can be found in Section S4.2, Supporting Information.

### Assessing the Thermodynamic (Dis)Advantages of Redox Systems:

With an easily accessible Gibbs free energy for each reactant/product in the CLAS cycles, the Gibbs reaction free energies (Δ*G*_r_) of one specific sub‐reaction in any chemical looping process can be calculated by

(10) $$\Delta G_{r}\left(T\right)=\sum_{i}^{\text{products}}\nu_{i}\Delta G_{\text{f,i}}\left(T\right)-\sum_{j}^{\mathrm{reactants}}\nu_{j}\Delta G_{\text{f,j}}\left(T\right)$$

where ν_*i*_ is the stoichiometric coefficient of species *i*. The optimal temperature (*T*_opt_) for one sub‐reaction was determined as it minimized the Δ*G*_r_(*T*) in a range of discrete numbers

(11) $$T_{\text{opt}}=\arg\min_{T}\Delta G_{r}\left(T\right),T=T_{0},T_{1},\text{…},T_{\max}$$

Therefore at *T*_opt_, the chemical equilibrium shifted to have the strongest tendency to form the products. Promising redox systems will be given by the pairs that led to the most negative Δ*G*_r_ in all individual steps, meaning that they allowed the spontaneous conversion within the entire cycle. The overall evaluation of each redox pair in entire CLAS cycle required a global descriptor that comprised the thermodynamic information from all correlated steps. The thermodynamic assessment was often referred as the “limiting reaction,” which can be defined as the step with the highest reaction energy. Considering an arbitrary CLAS process which carried out the *i*th sub‐reaction at *T*_i, opt_, the limiting reaction free energy can be denoted as

(12) $$\Delta G_{r,\lim}=\max\left\{\Delta G_{r_{1}}\left(T_{\text{1,opt}}\right),\Delta G_{r_{2}}\left(T_{\text{2,opt}}\right),\text{…},\Delta G_{r_{n}}\left(T_{\text{n,opt}}\right)\right\}$$

As such, whether a redox pair would enable spontaneous looping can be determined.

## Conflict of Interest

The authors declare no conflict of interest.

## Supporting information

Supporting Information

Supporting Information

## Data Availability

The data that support the findings of this study are openly available in Harvard Dataverse at https://doi.org/10.7910/DVN/4PG3R8 and https://doi.org/10.7910/DVN/EOOPKN, reference number 0.
