# Supplementary material for: High‐Throughput Screening of Bicationic Redox Materials for Chemical Looping Ammonia Synthesis
Source: Adv Sci (Weinh). 2022 Jul 24;9(27):2202811. doi: 10.1002/advs.202202811 (PMC9507380; doi:10.1002/advs.202202811)
Supplement: Supplementary file 1 — Supporting Information [file ADVS-9-2202811-s001.pdf]

Supporting Information: High-throughput Screening of Bicationic Redox Materials for Chemical Looping Ammonia Synthesis

*Jiaxin Fan Wenxian Li Sean Li Jack Yang**

J. Fan, Dr. W. Li, Prof. S. Li, Dr. J. Yang
Materials and Manufacturing Futures Institute, School of Material Science and Engineering, University of New South Wales, Sydney, NSW 2052, Australia
Email Address:jianliang.yang1@unsw.edu.au

Section S1 Chemical equations of four chemical looping processes

Here, We list out all the sub-reactions for the four CLs investigated in this work, namely the 2-Step/3-Step H_2O-CL, H_2-CL, and MH-CL. Here, all the reaction stoichiometries are balanced to yield one mole NH_3 per each cycle. It is worth noting that the compounds are not shared across different CLs, *e.g.* M_aO_b in H_2O-CL and M_aN_b in H_2-CL do not necessarily correspond to the same compound for a specific M. The colors to highlight redox materials and NH_3 in the chemical equations are consistent with Figure 1 in the main manuscript.

CLs driven by monocationic redox pairs (M–X) can be represented as:

2-Step H_2O-CL:

Rxn I. Nitrogen fixation:

$$\frac{c}{ad}M_aO_b + \frac{bc}{ad}H_2 + \frac{1}{2}N_2 \longrightarrow \frac{1}{d}M_cN_d + \frac{bc}{ad}H_2O$$

Rxn II. Hydrolysis:

$$\frac{1}{d}M_cN_d + \frac{bc}{ad}H_2O \longrightarrow \frac{c}{ad}M_aO_b + NH_3 + (\frac{bc}{ad} - \frac{3}{2})H_2$$

3-Step H_2O-CL:

Rxn I. Reduction of oxide:

$$\frac{c}{ad}M_aO_b + \frac{bc}{ad}H_2 \longrightarrow \frac{1}{d}M + \frac{bc}{ad}H_2O$$

Rxn II. Nitridation of metal:

$$\frac{1}{d}M + \frac{1}{2}N_2 \longrightarrow \frac{1}{d}M_cN_d$$

Rxn III. Hydrolysis:

$$\frac{1}{d}M_cN_d + \frac{bc}{ad}H_2O \longrightarrow \frac{c}{ad}M_aO_b + NH_3 + (\frac{bc}{ad} - \frac{3}{2})H_2$$

H_2-CL:

Rxn I. Reduction of N-rich nitride:

$$\frac{1}{b-c}M_aN_b + \frac{3}{2}H_2 \longrightarrow \frac{1}{b-c}M_aN_c + NH_3$$

Rxn II. Nitrogen fixation:

$$\frac{1}{b-c}M_aN_c + \frac{1}{2}N_2 \longrightarrow \frac{1}{b-c}M_aN_b$$

MH-CL:

Rxn I. Hydrogenation:

$$\frac{1}{b}M_aN_bH_c + (\frac{3}{2} + \frac{d-c}{2\,b})H_2 \longrightarrow \frac{1}{b}M_aH_d + NH_3$$

Rxn II. Nitridation of hydride:

$$\frac{1}{b}M_aH_d + \frac{1}{2}N_2 \longrightarrow \frac{1}{b}M_aN_bH_c + \frac{d-c}{2\,b}H_2$$

Similarly, CLs driven by bicationic redox pairs (M–M′–X) can be represented as:

2-Step H_2O-CL:

Rxn I. Nitrogen Fixation:

$$\frac{\varepsilon}{\theta}M_\alpha M'_\beta O_\gamma + \frac{\varepsilon\gamma}{\theta}H_2 + \frac{1}{2}N_2 \longrightarrow \frac{1}{\theta}M_{\varepsilon\alpha}M'_{\varepsilon\beta}N_\theta + \frac{\varepsilon\gamma}{\theta}H_2O$$

Rxn II. Hydrolysis:

$$\frac{1}{\theta}M_{\varepsilon\alpha}M'_{\varepsilon\beta}N_\theta + \frac{\varepsilon\gamma}{\theta}H_2O \longrightarrow \frac{\varepsilon}{\theta}M_\alpha M'_\beta O_\gamma + NH_3 + (\frac{\varepsilon\gamma}{\theta}-\frac{3}{2})H_2$$

3-Step H_2O-CL:

Rxn I. Reduction of oxide:

$$\frac{\varepsilon}{\theta}M_\alpha M'_\beta O_\gamma + \frac{\varepsilon\gamma}{\theta}H_2 \longrightarrow \frac{\varepsilon\alpha}{\theta}M + \frac{\varepsilon\beta}{\theta}M' + \frac{\varepsilon\gamma}{\theta}H_2O$$

Rxn II. Nitridation of two metals:

$$\frac{\varepsilon\alpha}{\theta}M + \frac{\varepsilon\beta}{\theta}M' + \frac{1}{2}N_2 \longrightarrow \frac{1}{\theta}M_{\varepsilon\alpha}M'_{\varepsilon\beta}N_\theta$$

Rxn III. Hydrolysis:

$$\frac{1}{\theta}M_{\varepsilon\alpha}M'_{\varepsilon\beta}N_\theta + \frac{\varepsilon\gamma}{\theta}H_2O \longrightarrow \frac{\varepsilon}{\theta}M_\alpha M'_\beta O_\gamma + NH_3 + (\frac{\varepsilon\gamma}{\theta}-\frac{3}{2})H_2$$

H_2-CL:

Rxn I. Reduction of N-rich nitride:

$$\frac{\varepsilon}{\varepsilon\gamma-\delta}M_\alpha M'_\beta N_\gamma + \frac{3}{2}H_2 \longrightarrow \frac{1}{\varepsilon\gamma-\delta}M_{\varepsilon\alpha}M'_{\varepsilon\beta}N_\delta + NH_3$$

Rxn II. Nitrogen fixation:

$$\frac{1}{\varepsilon\gamma-\delta}M_{\varepsilon\alpha}M'_{\varepsilon\beta}N_\delta + \frac{1}{2}N_2 \longrightarrow \frac{\varepsilon}{\varepsilon\gamma-\delta}M_\alpha M'_\beta N_\gamma$$

MH-CL:

Rxn I. Hydrogenation:

$$\frac{\varepsilon}{\gamma}M_\alpha M'_\beta N_\gamma H_\theta + (\frac{3}{2} + \frac{\mu-\theta}{2\,\gamma})H_2 \longrightarrow \frac{1}{\gamma}M_{\varepsilon\alpha}M'_{\varepsilon\beta}H_\mu + NH_3$$

Rxn II. Nitridation of hydride:

$$\frac{1}{\gamma}M_{\varepsilon\alpha}M'_{\varepsilon\beta}H_\mu + \frac{1}{2}N_2 \longrightarrow \frac{\varepsilon}{\gamma}M_\alpha M'_\beta N_\gamma H_\theta + \frac{\mu-\theta}{2\,\gamma}H_2$$

Section S2 Workflow of retrieving data from Materials Project

The material information is retrieved via Materials Project's in-built API[1] from the database with version of 2021.11.10. Several selection criteria are applied to remove the unsuitable materials. Only the most stable structures for each chemical compositions in the database, *i.e.* ones have the lowest formation energies [2], are considered as the screening candidates in the thermochemical reactions.

For monocationic system, the total number of elements is set to be 2. One element is limited to be the associated anion in the active material while another cation is from a group of elements that has the atomic number up to 83 (Bi). These cation elements range from alkali/akaline-earth metals, transition metals, and post-transition metals, metalloids, as well as some non-metal elements P, S, Se. To be consistent with the previous high-throughput study [3], the total number of atoms in the crystal unit cells of oxides and nitrides is set to be less than 20. Additionally, the coefficients of H_2 ($\frac{ad}{bc} - \frac{3}{2}$ and $\frac{\theta}{\varepsilon\gamma} - \frac{3}{2}$) in Equations 2 and 4 (Section S1) are set to be non-negative to restrain the reactants between nitrides and steam in hydrolysis process. Matching the redox pairs is based on the stoichiometries in the reaction cycles illustrated in Section S1.

Similarly, for bicationic systems, compounds that contain two cations and have less than 30 atoms in the unit cell are selected as candidates in this study. Comparing to monocationic materials, although there are significantly more bicationic compounds existed in the database, the number of matched pairs is surprisingly not much larger as shown in Table 1. It is mainly owing to the strict precondition that the stoichiometries of the two cations need to be proportional in the commensurate materials, which restricts the number of redox pairs that could be applied in the thermochemical cycle.

The raw data supporting the findings of this study can be openly accessed from `https://dataverse.harvard.edu/dataverse/jackyang-unsw`. The Python codes for constructing the chemical looping processes and calculating the Gibbs energies are available at `https://github.com/yangjackie/futuremat_public`.

Section S3 Selecting the x-axis for the volcano plots

The Gibbs free energies at elevated temperatures are derived from the ground-state ΔH_f with the additional entropy contribution that is accounted for by the Gibbs free energy descriptor (Equation 8). Therefore, an assessment of the correlation between $\Delta G_\mathrm{r,lim}$ and ΔH_f of the redox pairs would help to facilitate the identifications of the most viable pairs of active materials. Figure S6 demonstrates the change of $\Delta G_\mathrm{r,lim}$ distribution when plotting against either the ΔH_f of individual active materials or their energy difference for the 2-Step and 3-Step H_2O-CL. The $\Delta G_\mathrm{r,lim}$-ΔH_f plots show a clear volcano dependency only when $\Delta H_\mathrm{f,\ oxd}$ is selected for 3-Step H_2O-CL (left panel in Figure S5a) and the formation enthalpy difference between two redox materials for the 2-Step cycles ($\Delta H_\mathrm{f,\ oxd} - \Delta H_\mathrm{f,\ nit}$, right panel of Figure S5b). Apparent division of limiting reactions can also be observed, which gives unique insights of the correlation between phase stability of active materials and CL spontaneity. Changing which enthalpy information is presented in the x-axis would often mask the volcano-type dependence and overlap the redox pairs with different types of limiting reactions.

This selectivity of energy feature can be intrinsically attributed to the nature of the sub-reactions in looping processes. Because the reduced and oxidized forms of a redox pair coexist in both sub-reactions in cycles with only two steps (2-Step H_2O-CL and H_2-CL), it makes ΔG_r to be more sensitive to the relative formation enthalpies of the redox pairs. Thus it is a better representation of the global $\Delta G_\mathrm{r,lim}$ compared to only using one of the ΔH_f in the redox pairs.[3] In contrast, the Rxn I and II in 3-Step H_2O-CL involve the third intermediate metallic state of which ΔH_f is defined to be 0 in the calculation of ΔG_r in Equation 10. Therefore in this case, a single ΔH_f is sufficient to reflect this energy correlation.

Section S4 Benchmark of the machine learning models with first-principles calculations

Section S4.1 Physical descriptor for Gibbs energy calculation at elevated temperature

To test the performance of the Gibbs energy descriptor, a benchmarking calculation was conducted by using the quasi harmonic approximation (QHA) module[4, 5] in the PHONOPY code[6]. We selected 33 bicationic compounds from MP, which comprise 15 oxides, 10 nitrides, 4 hydrides, and 4 nitride hydrides. These materials have a wide coverage of various cation combinations and space groups, of which the detailed information can be found in Table S2.

The electronic structures were carried out by the *Vienna Ab initio Simulation Package* (VASP)[7, 8]. The electronic wavefunctions were expanded using the projector augmented wave (PAW) method and Perdew–Burke-Ernzerhof (PBE) exchange–correlation functional was applied. To ensure the interatomic forces have been fully relaxed after structure optimization, the global break conditions for the electronic self-consistent loop and ionic relaxation loop (i.e. EDIFF and EDIFFG tags in INCAR) were set to be 10^{-8} eV and 10^{-8} eV Å^{-1} respectively while the reciprocal space resolution of k-points for each structure is set to be 0.05. $2 \times 2 \times 2$ supercells with a finite atomic displacement of 0.01 Å were generated for most of the QHA calculations. Larger supercell dimensions were sometimes chosen when the effect of the self-interaction of displaced atoms cannot be neglected in the small cells.

The calculated Gibbs energies from 300 – 1800 K were plotted in Figure S13. The mean absolute errors (MAEs) of oxides, nitrides, hydrides, and nitride hydrides are 64 meV atom^{-1}, 66 meV atom^{-1}, 82 meV atom^{-1}, and 21 meV atom^{-1} respectively. These values are comparable to the reported model accuracy in the original literature.[9]

Section S4.2 Formation energy prediction using BOWSR and MEGNet

We tested the collective performance of BOWSR and MEGNet algorithm with a variety of structures acquired from MP. We compared the MEGNet-predicted formation energies of the optimized structures with the DFT-calculated values in the database. The benchmarking dataset has 688 structures with non-repetitive chemical compositions, which consists of binary/ternary hydrides (M–H/M_1–M_2–H) and ternary/quaternary nitride-hydrides (M–N–H/M_1–M_2–N–H) with the lowest formation energies in their chemical spaces.

Figure S6a compared the predicted formation energies from machine learning model to the DFT results with the threshold of the atomic distance set as 1 Å. The MAEs of M_1–M_2–H and M–N–H/M_1–M_2–N–H are 66.80 meV atom^{-1}, 45.67 meV atom^{-1}, and 51 meV atom^{-1} respectively, which all fall below the average model accuracy of 88 meV atom^{-1}.[10] Only the MAE of binary hydrides is 162.48 meV atom^{-1}, which significantly exceeds the reported average value. It can be largely attributed to the structural instability of binary hydrides ($\Delta H_{\mathrm{f}} >= 0$) and the general challenge in relaxing structures with small and light elements. The influence of the cutoff distance is examined with respect to the MAEs in Figure S6b to ensure 1 Å is a rational option. It also helps to prevent the optimized crystal structures from having unreasonably large or small interatomic distance. The Gaussian-like distribution of the errors is plotted in Figure S6c.

Section S5 Figures and Tables

Redox material (pair)	CL process	Exp. application
$FeMoO_4/Fe_3Mo_3N$	H_2O-CL	NRR[11, 12, 13, 14]
$CoWO_4/Co_3W_3N$	H_2O-CL	OER/ORR[15, 16]
$LiCoO_2$	H_2O-CL	OER[17]
$BaNiO_3$	H_2O-CL	OER[18], dry reforming methane[19]
Mn_2CuO_4	H_2O-CL	Electrochemical H_2O_2 sensing[20]
$NaFeO_2$	H_2O-CL	Biodiesel synthesis[21], Co/CO2 sorption[22]
$LiNiO_2$	H_2O-CL	OER[23]
$SrNiO_3$	H_2O-CL	Dry reforming of propane[24],methane reforming[25]
Mn_2NiO_4	H_2O-CL	Reduction of nitrogen oxides[26]
Li_2IrO_3	H_2O-CL	OER[27]
$Cu_2P_2O_7$	H_2O-CL	CO_2 reduction[28]
$MoWO_6$	H_2O-CL	OER[29]
Mn-Na-H-N	MH-CL	Ammonia synthesis[30]
Li-Pd-H-N	MH-CL	Ammonia synthesis[31]

Table S1: Redox materials of which catalytic performances have been experimentally studied in literature. NRR, OER, and ORR refer to nitrogen reduction reaction, oxygen evolution reaction, and oxygen reduction reaction respectively.

MP id	Formula	Type	Supercell	Space group
mp-1068011	$SrAlH_3$	hydrides	3 3 3	Pm-3m
mp-1068746	$TlHPd_3$	hydrides	3 3 3	Pm-3m
mp-1227045	CaH_2Pd	hydrides	3 3 3	P4/mmm
mp-28797	YHSe	hydrides	3 3 3	P-6m2
mp-1103502	$CaB_2(H_5N)_2$	nit-hydrides	2 2 2	C2
mp-1193106	$Li_2Mg(HN)_2$	nit-hydrides	2 2 2	Pbcn
mp-642740	$CsLi(H_2N)_2$	nit-hydrides	2 2 2	$P6_222$
mp-643359	$Rb_2Sn(H_2N)_6$	nit-hydrides	2 2 2	P-3
mp-1029791	$MgSnN_2$	nitrides	2 2 2	$Pna2_1$
mp-1216767	$TiVN_2$	nitrides	2 2 2	R-3m
mp-1217820	$TaVN_2$	nitrides	2 2 2	P4/mmm
mp-1245552	$TiFeN_2$	nitrides	2 2 2	Pnma
mp-1246072	$Y_2Mn_3N_4$	nitrides	2 2 2	C2/c
mp-1246497	Li_2IrN_2	nitrides	2 2 2	P-3m1
mp-1246669	$MgCoN_2$	nitrides	2 2 2	$Pna2_1$
mp-1247032	$TiCoN_2$	nitrides	2 2 2	Pnma
mp-1247124	Li_2CoN_2	nitrides	2 2 2	P-3m1
mvc-15736	$Mg(WN)_2$	nitrides	2 2 2	P-4m2
mp-1178424	$CoNiO_3$	oxides	2 2 2	R-3
mp-1186154	$NaNiO_3$	oxides	2 2 2	Pm-3m
mp-1221950	$MgMn_2O_3$	oxides	3 3 2	P-3m1
mp-1221972	$MgSiO_4$	oxides	2 2 2	P2/m
mp-1245999	$ZnOsN_2$	oxides	2 2 2	$Pna2_1$
mp-18725	$YCrO_3$	oxides	2 2 2	Pnma
mp-18949	$VFeO_4$	oxides	2 2 2	Cmcm
mp-19082	$TiMnO_3$	oxides	2 2 2	R-3
mp-761472	Co_2CuO_4	oxides	2 2 2	Imma
mp-774472	PrSO	oxides	2 2 2	Cmce
mp-8189	$LiReO_3$	oxides	2 2 2	R3c
mp-8324	Dy_2S_2O	oxides	2 2 2	$P2_1/c$
mp-9158	$LiCuO_2$	oxides	2 2 2	C2/m
mvc-3999	$Ca_2Mo_2O_5$	oxides	2 2 2	Pbam
mvc-8344	$MgMn_2O_5$	oxides	2 2 2	Cmcm

Table S2: Materials selected for QHA calculations.

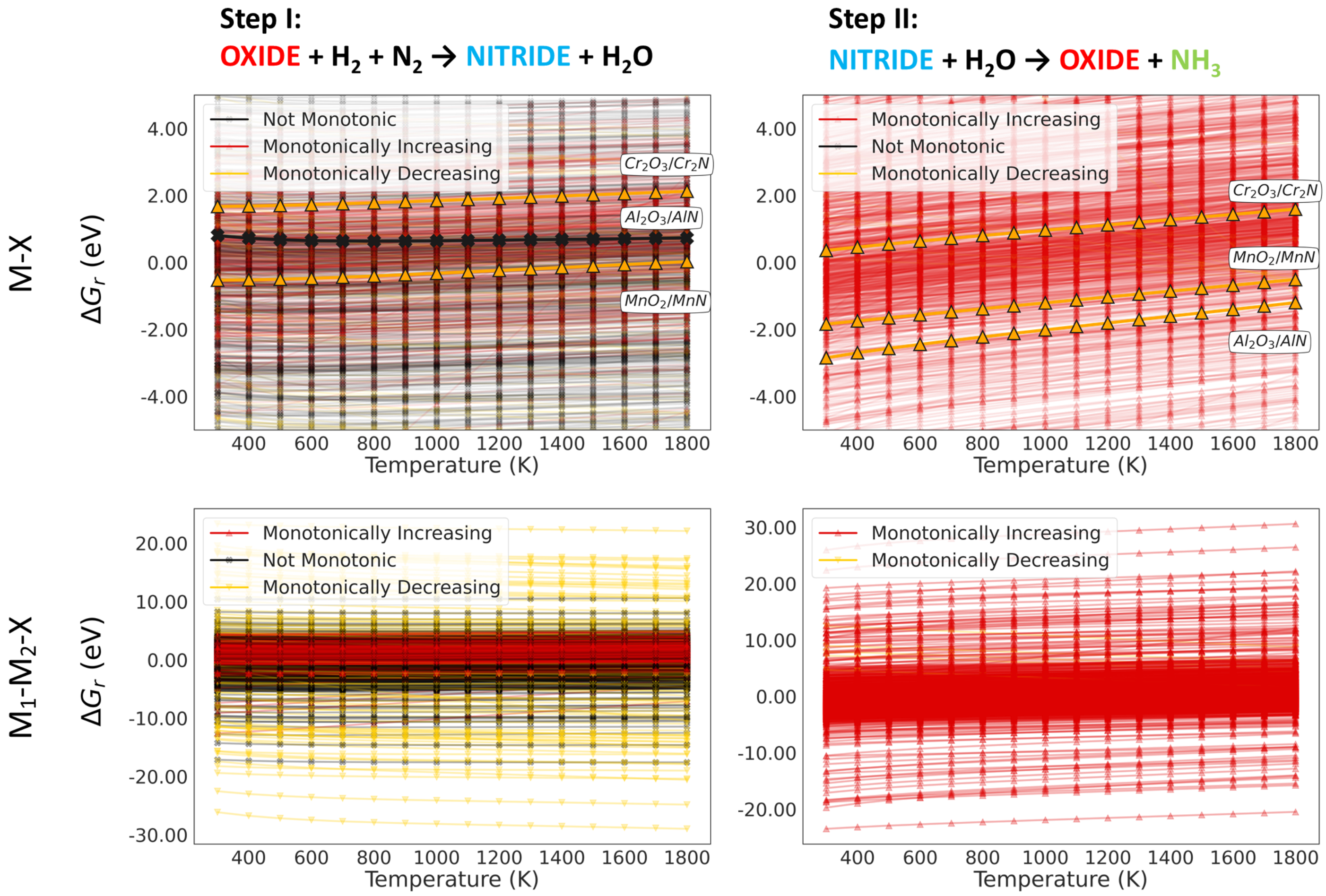

G_r Change	M-X		M_1-M_2-X	
	Step I	Step II	Step I	Step II
Increasing	**536**	**1161**	**858**	**1521**
Decreasing	371	12	541	5
Not Monotonic	267	1	127	0
Total	1174		1526	

Figure S1: Gibbs reaction energies (ΔG_r) of 1174 monocationic and 1526 bicationic oxide/nitride pairs calculated for 2-Step H_2O-CL. The ΔG_r are calculated between 300 K and 1800 K for the both sub-reactions. The red and yellow lines indicate the changes of ΔG_r are monotonically increasing or decreasing with respect to the rising temperature, while the redox pairs marked by black do not show monotonic relation. Monocationic redox pairs that have been investigated experimentally in literature are also highlighted (plots in the upper panel). The table below provides a more detailed summary of the numbers of redox pairs in each category.

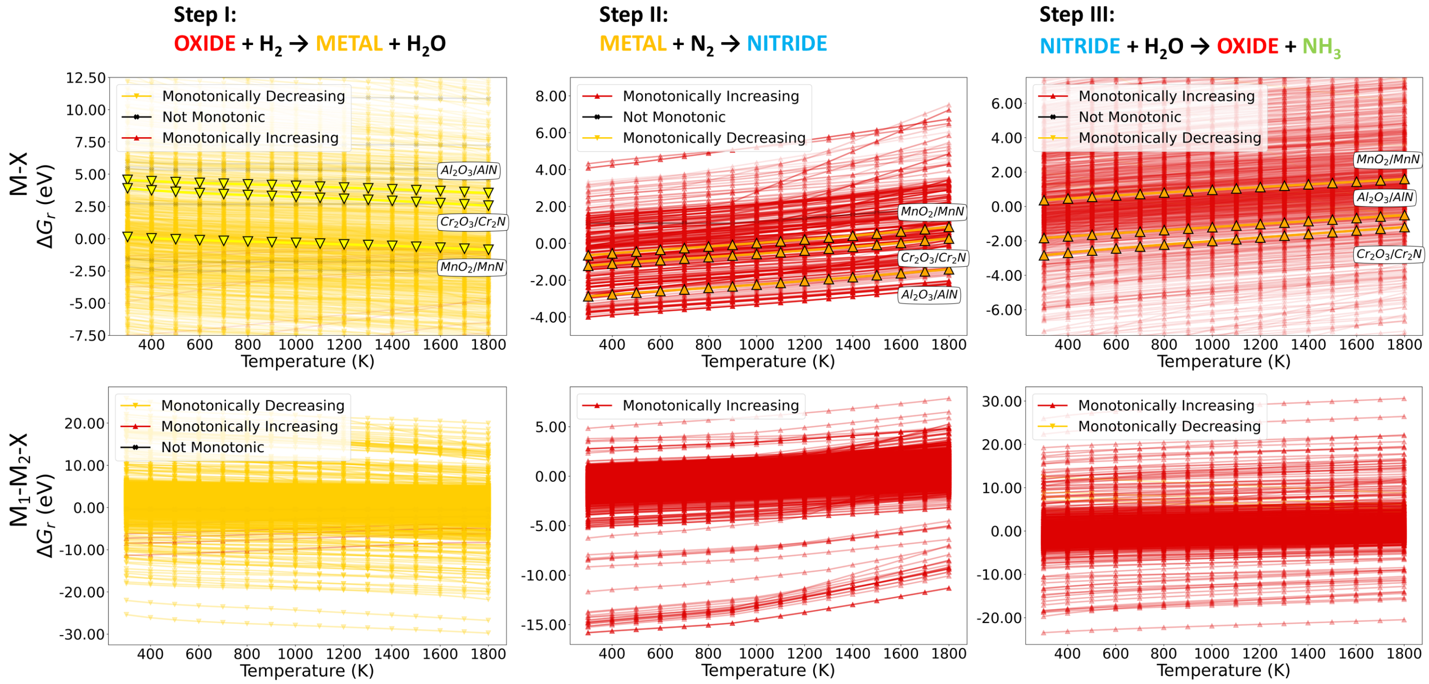

G_r Change	M-X			M_1-M_2-X		
	Step I	Step II	Step III	Step I	Step II	Step III
Increasing	12	**1164**	**1161**	5	**1526**	**1521**
Decreasing	**1151**	4	12	**1520**	0	5
Not Monotonic	11	6	1	1	0	0
Total	1174			1526		

Figure S2: Gibbs reaction energies (ΔG_r) of 1174 monocationic and 1526 bicationic oxide/nitride pairs calculated for 3-Step H_2O-CL. The ΔG_r are calculated between 300 K and 1800 K for the both sub-reactions. The red and yellow lines indicate the changes of ΔG_r are monotonically increasing or decreasing with respect to the rising temperature, while the redox pairs marked by black do not show monotonic relation. Monocationic redox pairs that have been investigated experimentally in literature are also highlighted (plots in the upper panel). The table below provides a more detailed summary of the numbers of redox pairs in each category.

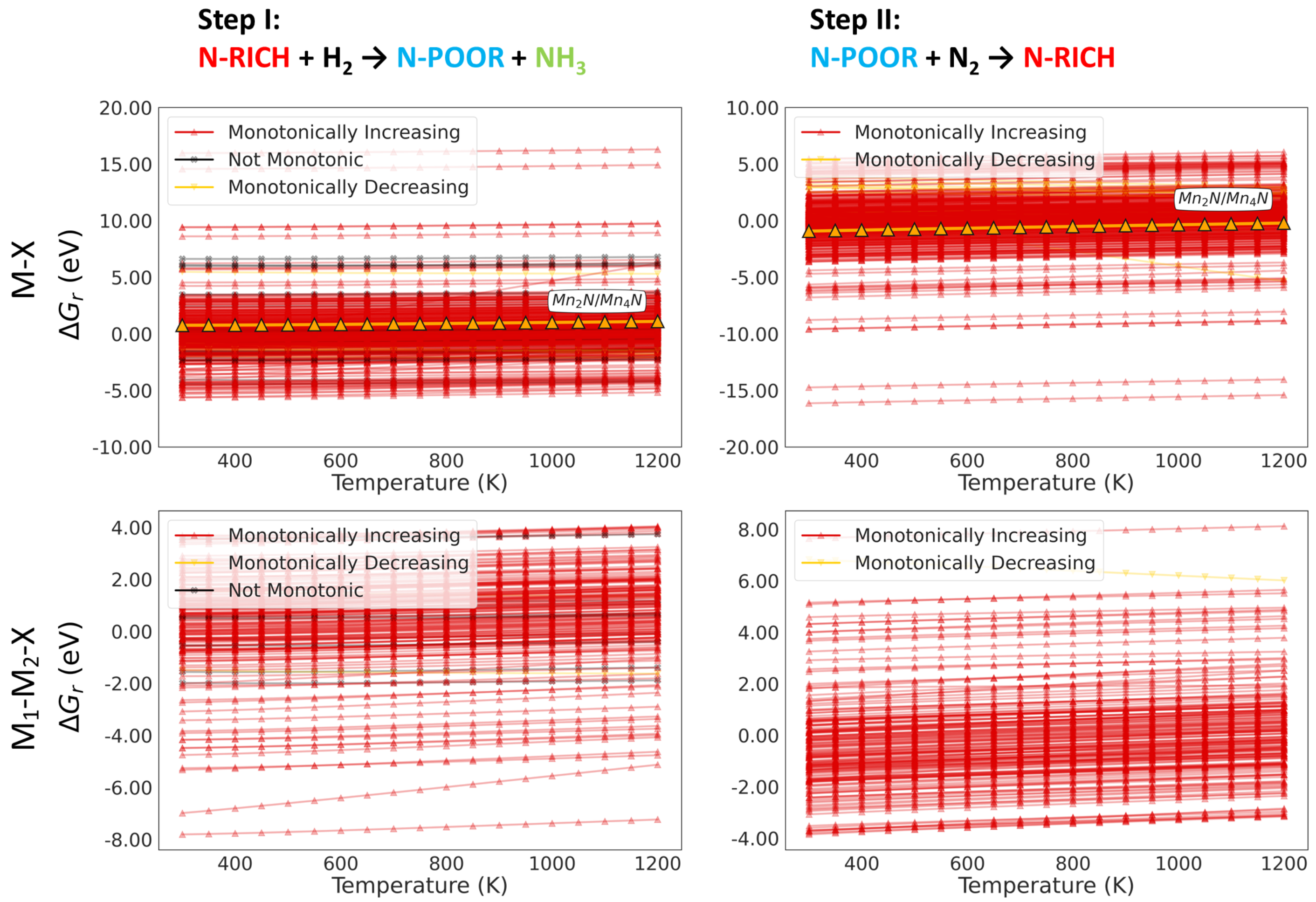

G_r Change	M-X		M_1-M_2-X	
	Step I	Step II	Step I	Step II
Increasing	**441**	**463**	**162**	**172**
Decreasing	7	10	2	1
Not Monotonic	25	0	9	0
Total	473		173	

Figure S3: Gibbs reaction energies (ΔG_r) of 473 monocationic and 173 bicationic nitrogen-rich/nitrogen-poor pairs calculated for H_2-CL. A lower maximum temperature of 1200 K is chosen in this calculation compared to H_2O-CL. The red and yellow lines indicate the changes of ΔG_r are monotonically increasing or decreasing with respect to the rising temperature, while the redox pairs marked by black do not show monotonic relation. The table below provides a more detailed summary of the numbers of redox pairs in each category.

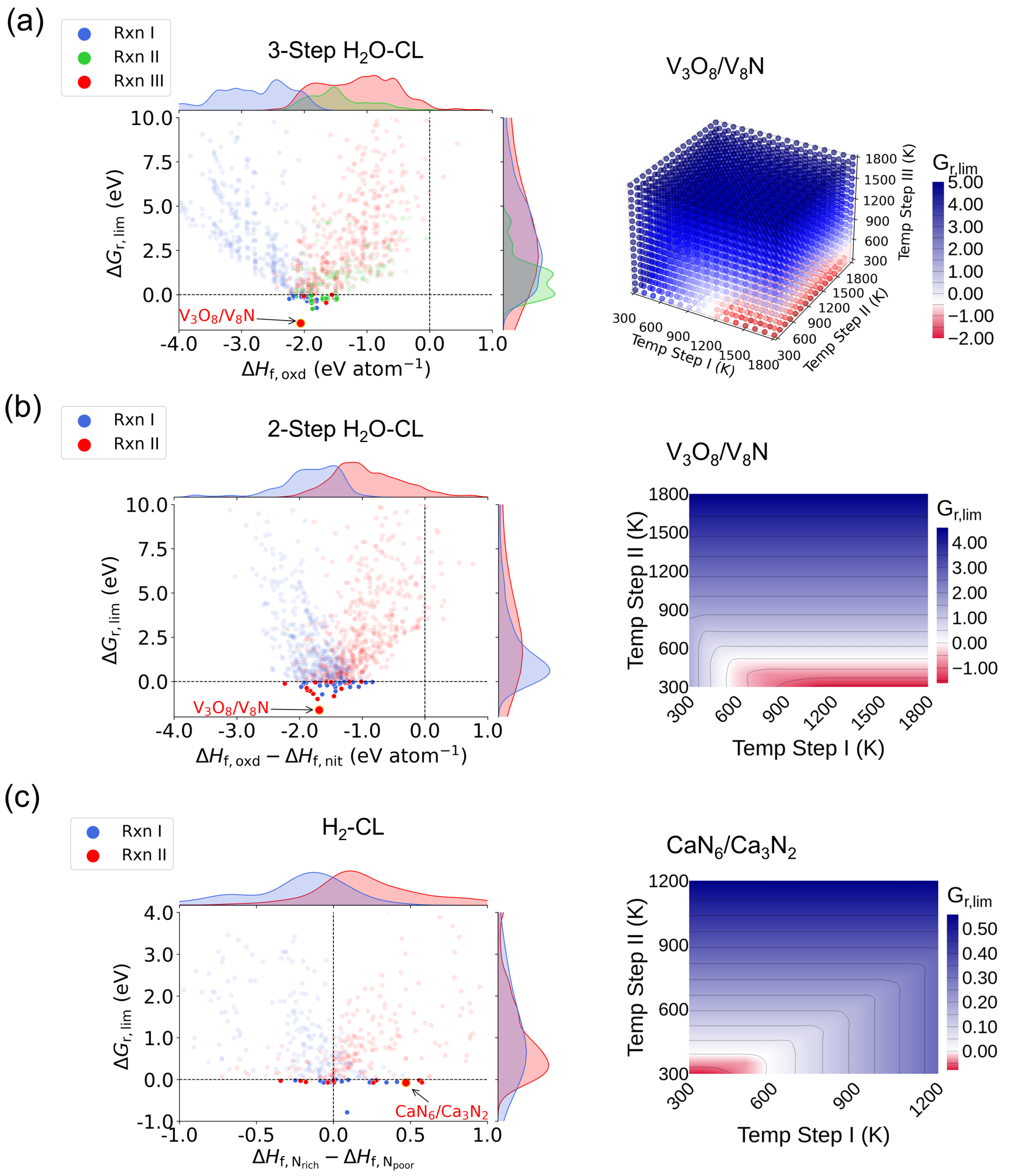

Figure S4: Volcano plots of the limiting energies using monocationic redox materials (M–X) in a) 3-Step H_2O-CL, b) 2-Step H_2O-CL, and c) H_2-CL. V_3O_8/V_8N has the most negative $\Delta G_{\mathrm{r,lim}}$ in both 2-Step and 3-Step H_2O-CL, of which the temperature dependence of $\Delta G_{\mathrm{r,lim}}$ was visualized as contour plots on the right panel of (a) and (b). S_3N_2/S_2N has the most negative $\Delta G_{\mathrm{r,lim}}$ for H_2-CL, of which the nitride phases are highly unstable. Hence CaN_6/Ca_3N_2 is instead highlighted as being the next best pair. The limiting reactions are marked as blue, green, and red for Rxn I, II, and III in the 3-Step H_2O-CL and as blue and red for Rxn I and Rxn II in the rest 2 step processes respectively (b and c).

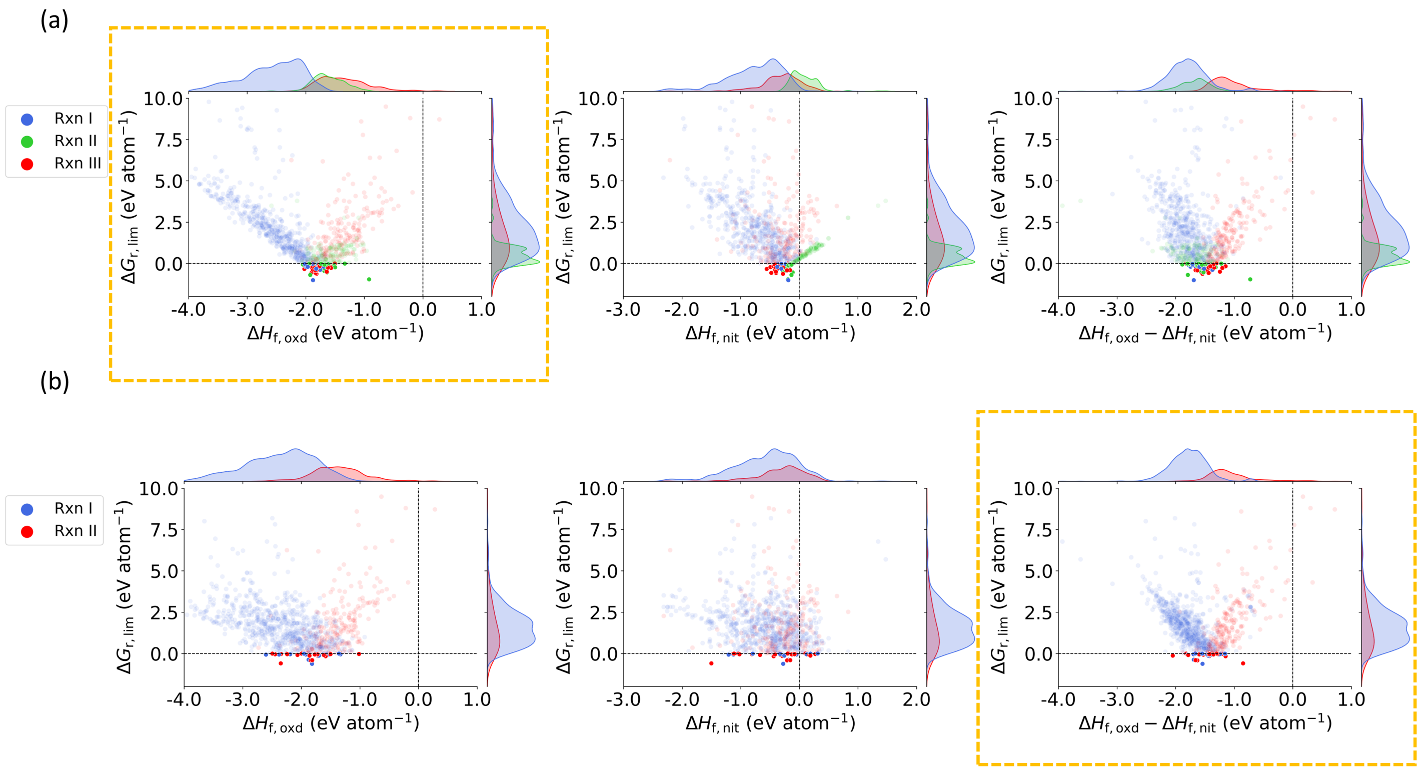

Figure S5: Limiting energies of M_1–M_2–N/O plotted against different formation energies of redox pairs for a) 3-Step H_2O-CL and b) 2-Step H_2O-CL. Highlighted plots are selected in the main manuscript in Figure 4.

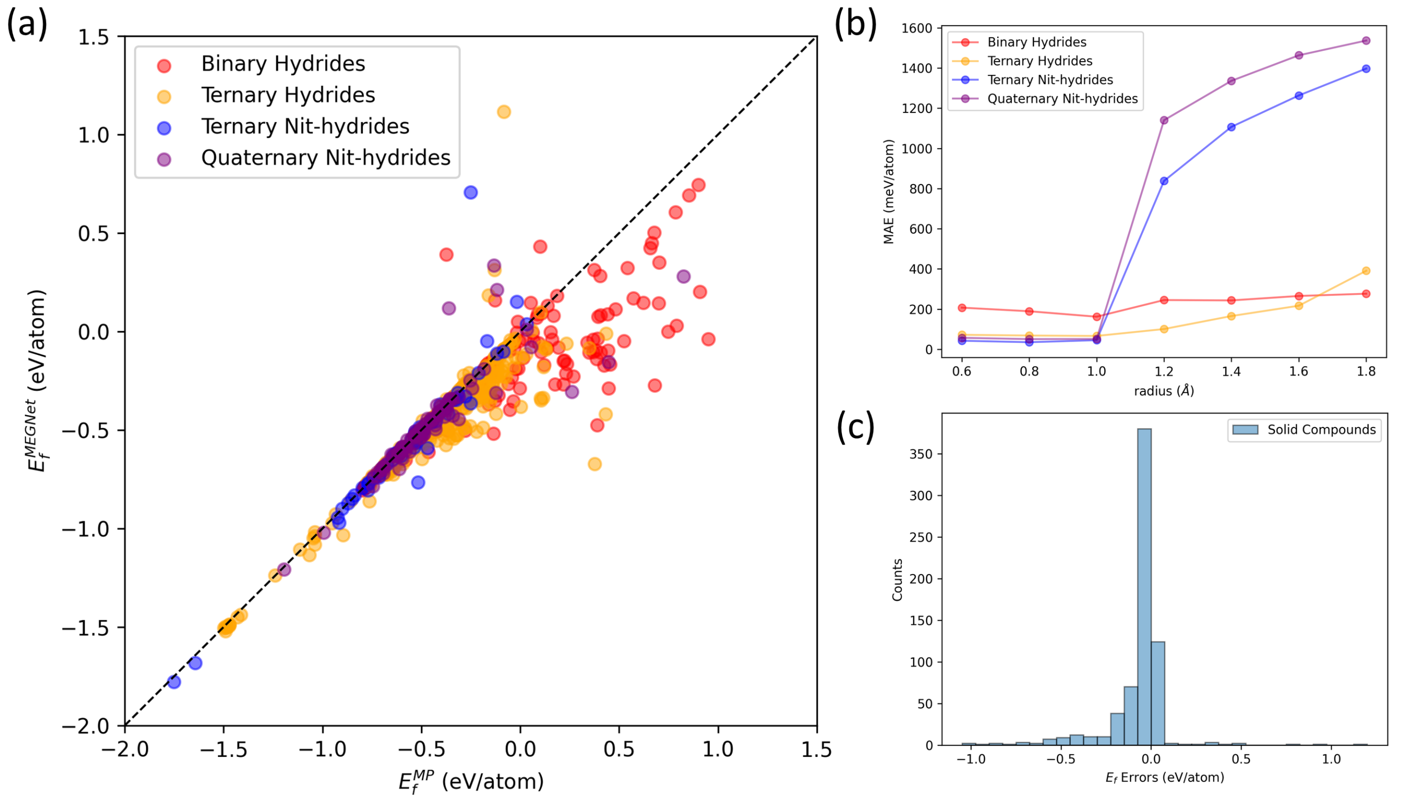

Figure S6: Benchmarking the performance of BOWSR and MEGNet in predicting formation energies of M−H, M_1−M_2−H, M−N−H, and M_1−M_2−N−H.

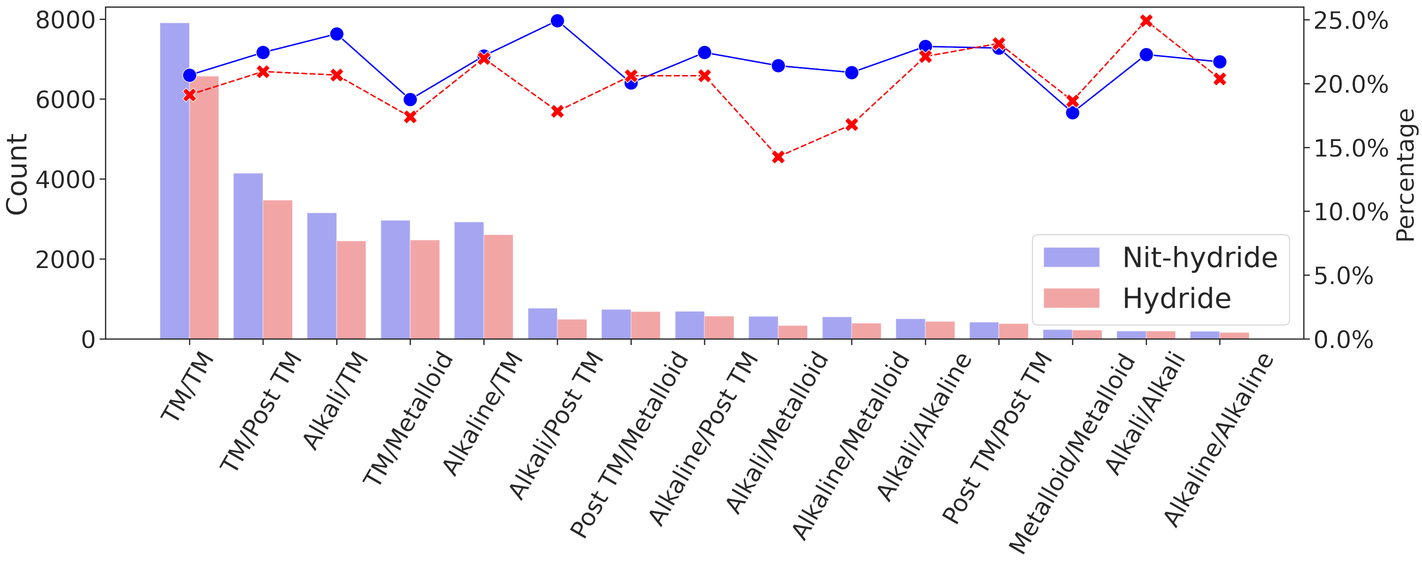

Figure S7: Numbers (bar plot) of viable candidates categorized by cation groups and their percentages (line plot) in all hypothetical compounds.

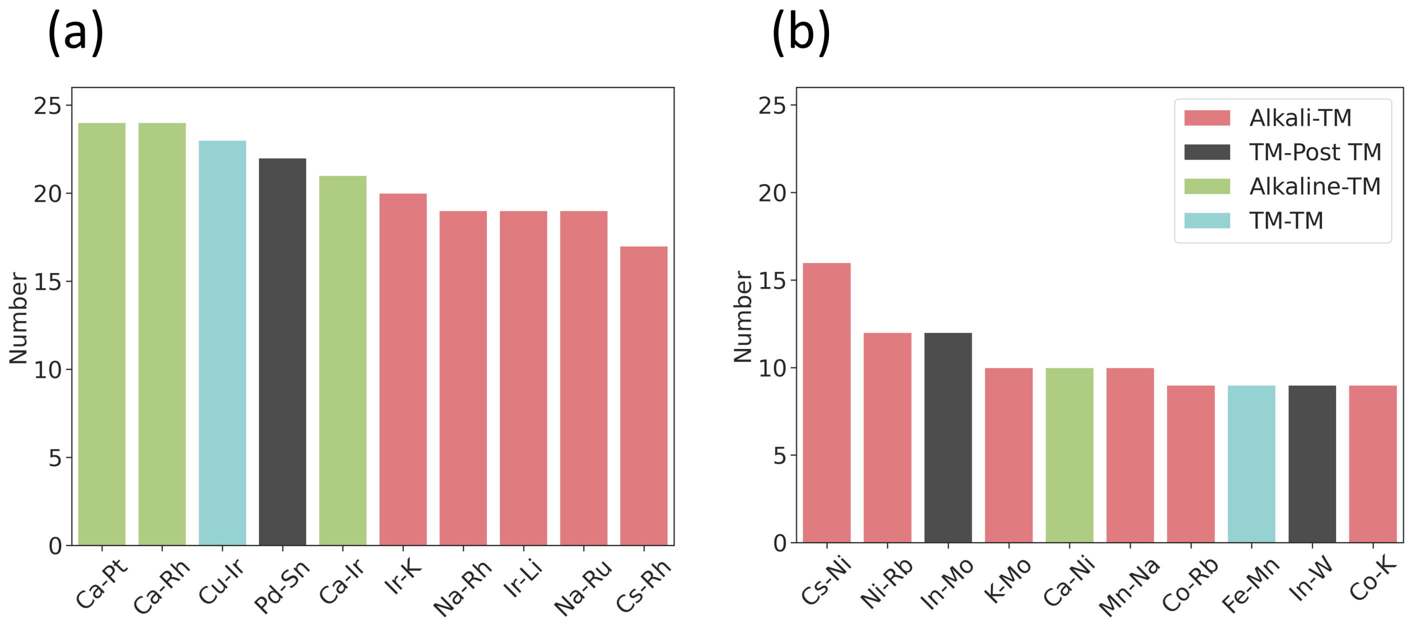

Figure S8: Occurrence of top 10 cation combinations for a) all viable redox pairs and b) redox materials excluding precious, toxic and radioactive elements.

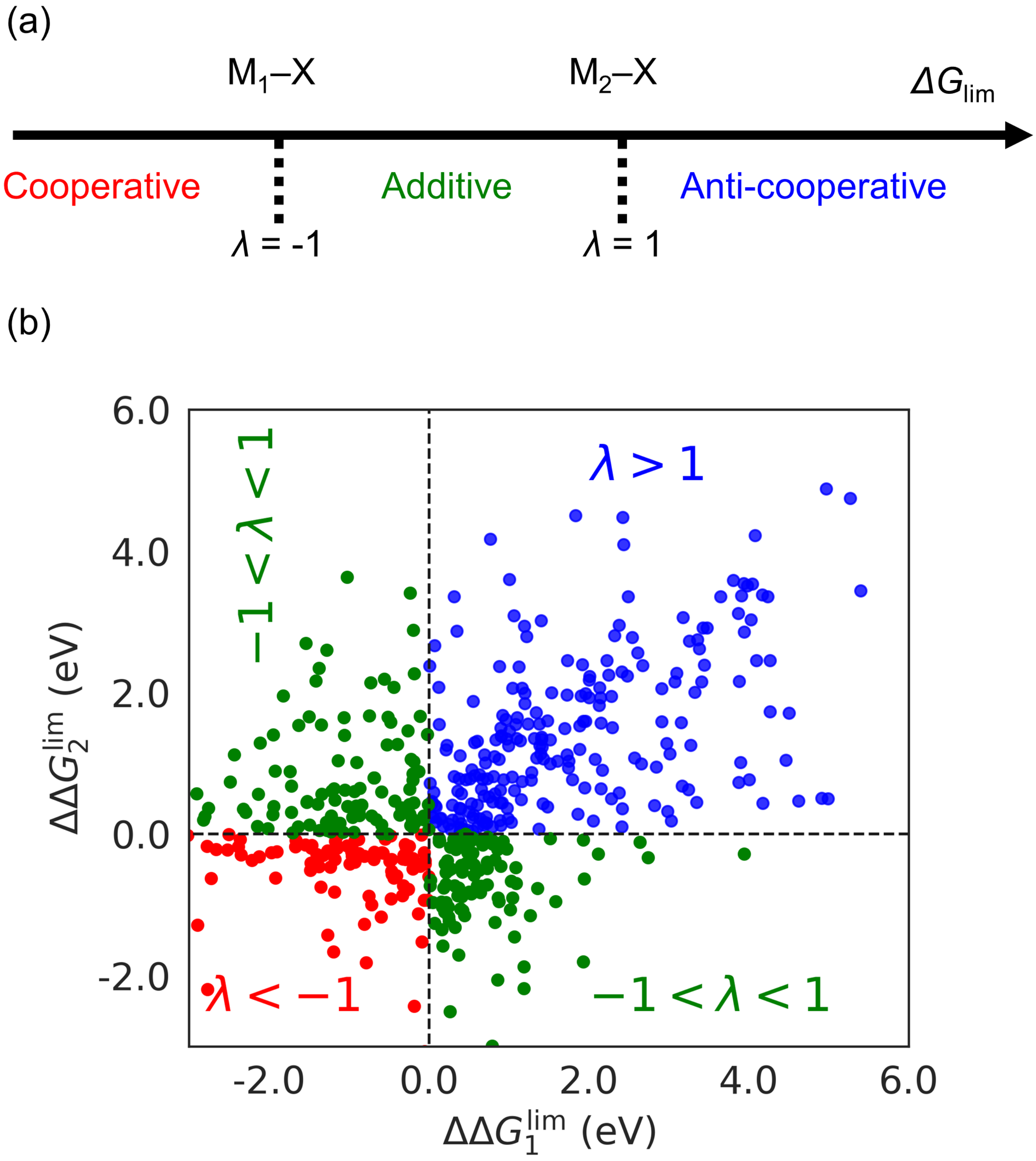

Figure S9: The relationship between λ metric and (a) $\Delta G_{\mathrm{r,lim}}$ and (b) $\Delta\Delta G_{\mathrm{i}}^{\mathrm{lim}}$.

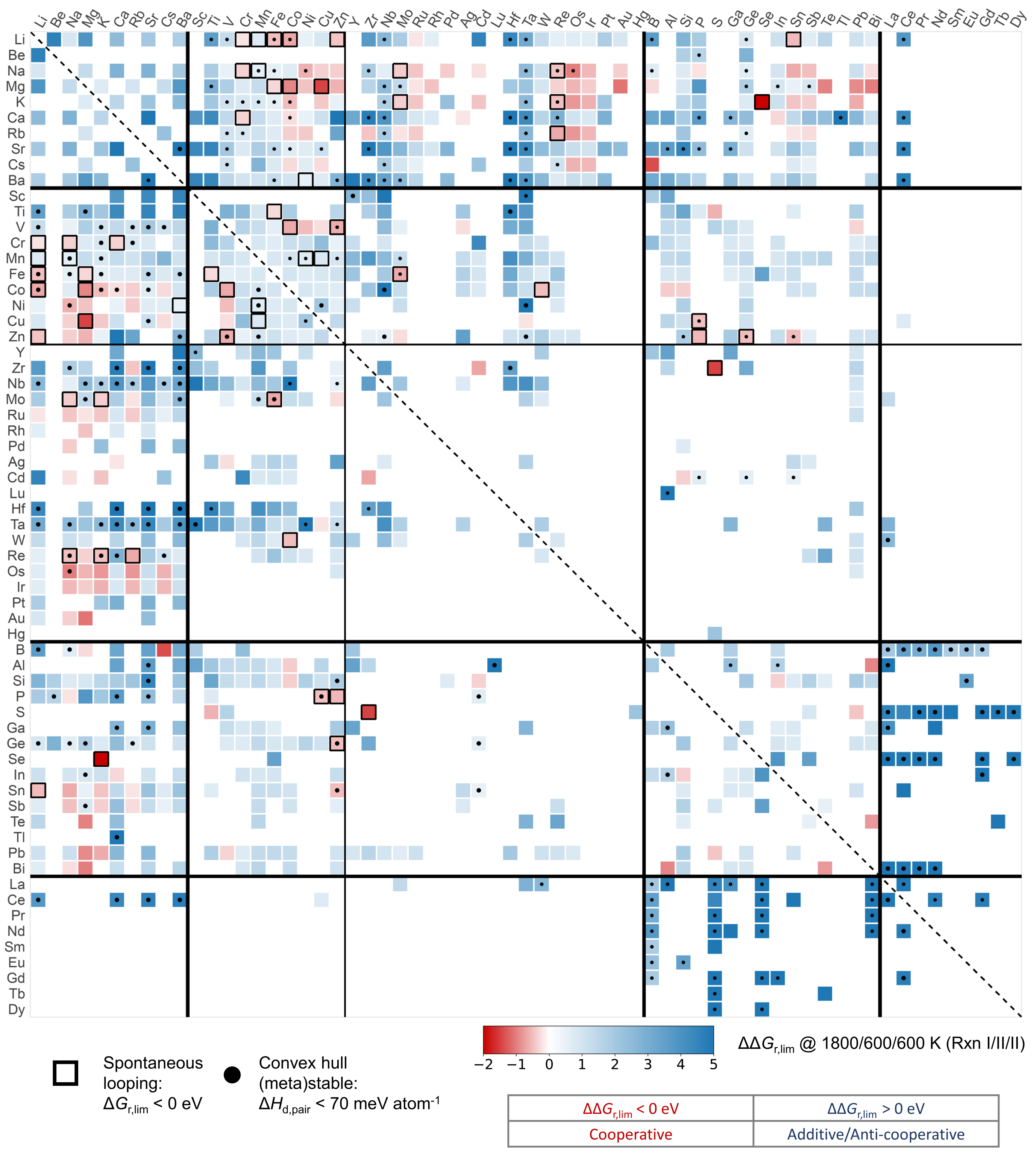

Figure S10: Heatmap of the differences in limiting energies ($\Delta\Delta G_{r,lim}$) between bicationic and monocationic redox pairs for 3-Step H_2O-CL. Blue color indicates monocationic pairs are more favorable than the bicationic ones where the opposite cases are indicated by the red color. The elements are grouped by their positions in the periodic table, *i.e.* *s*-block (alkali/alkaline earth metals), *d*-block (transition metals), *p*-block (post-transition metals, metalloids, and non-metals), and 4*f*-block. 3*d*-transition metals are further separated because of their highest rate of cooperative enhancement, convex hull (meta)stability, and looping spontaneity. Viable pairs with negative limiting energies are labelled by black squares. (Meta)stable active materials are marked by black dots.

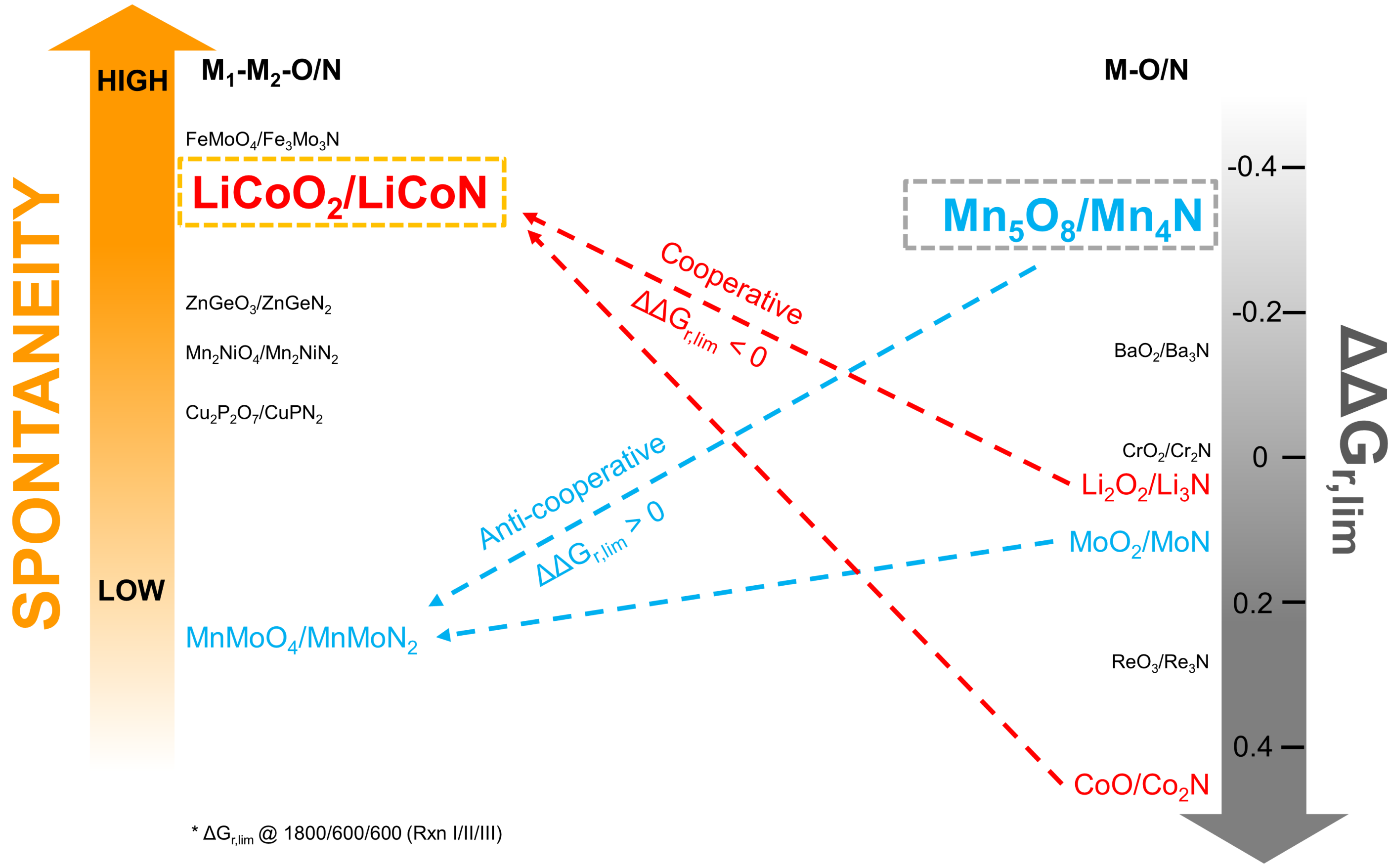

Figure S11: Illustration of (anti)cooperativity between M_1–M_2–O/N and M–O/N. Pairs colored as red demonstrate the cooperative effect, *i.e.* $\Delta\Delta G_{\mathrm{r,lim}} < 0$ whereas pairs in blue demonstrate the anti-cooperative effect with $\Delta\Delta G_{\mathrm{r,lim}} > 0$.

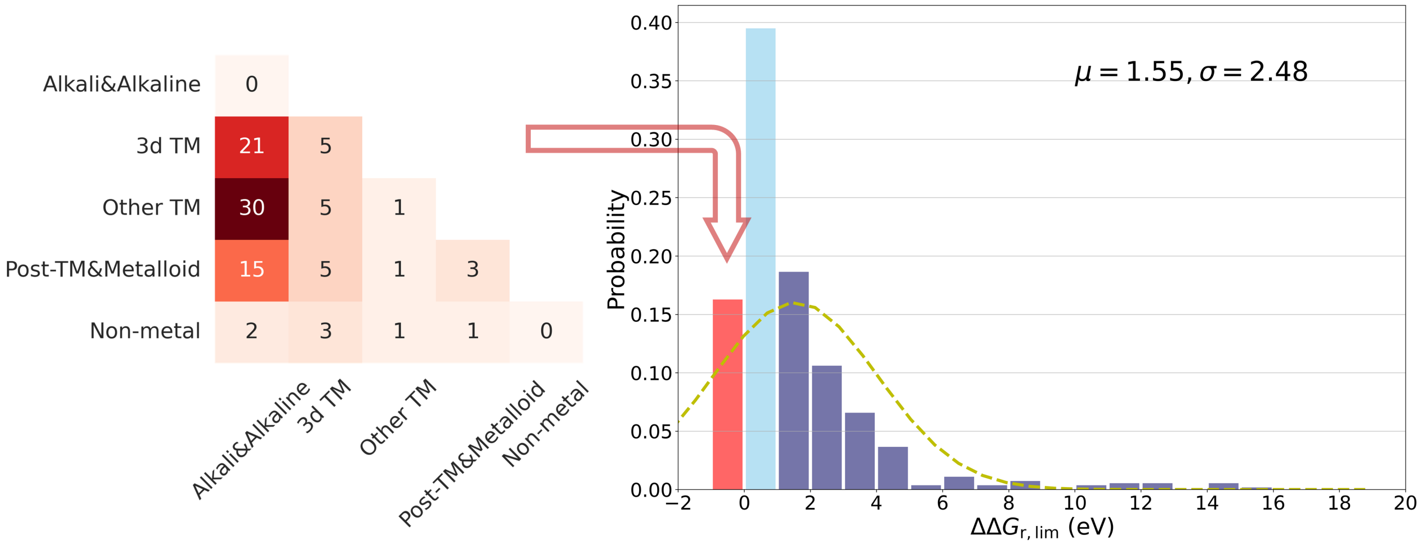

Figure S12: Statistical distribution of limiting energy differences between bicationic and monocationic pairs. Left panel: elements are grouped into alkali/alkaline earth metals, first transition metals (3d transition metals), other transition metals, post-transition metals/metalloids, and non-metals to count and classify the combinations. Right panel: bins marked in blue refer to the ternaries showing anti-cooperative effect in which $\Delta\Delta G_{\mathrm{r,lim}} > 1$ is marked in dark blue and $0 < \Delta\Delta G_{\mathrm{r,lim}} < 1$ (weak anti-cooperative) is marked in light blue. Red bin refers to cases that ternaries thermodynamically outperform binaries with $\Delta\Delta G_{\mathrm{r,lim}} < 0$.

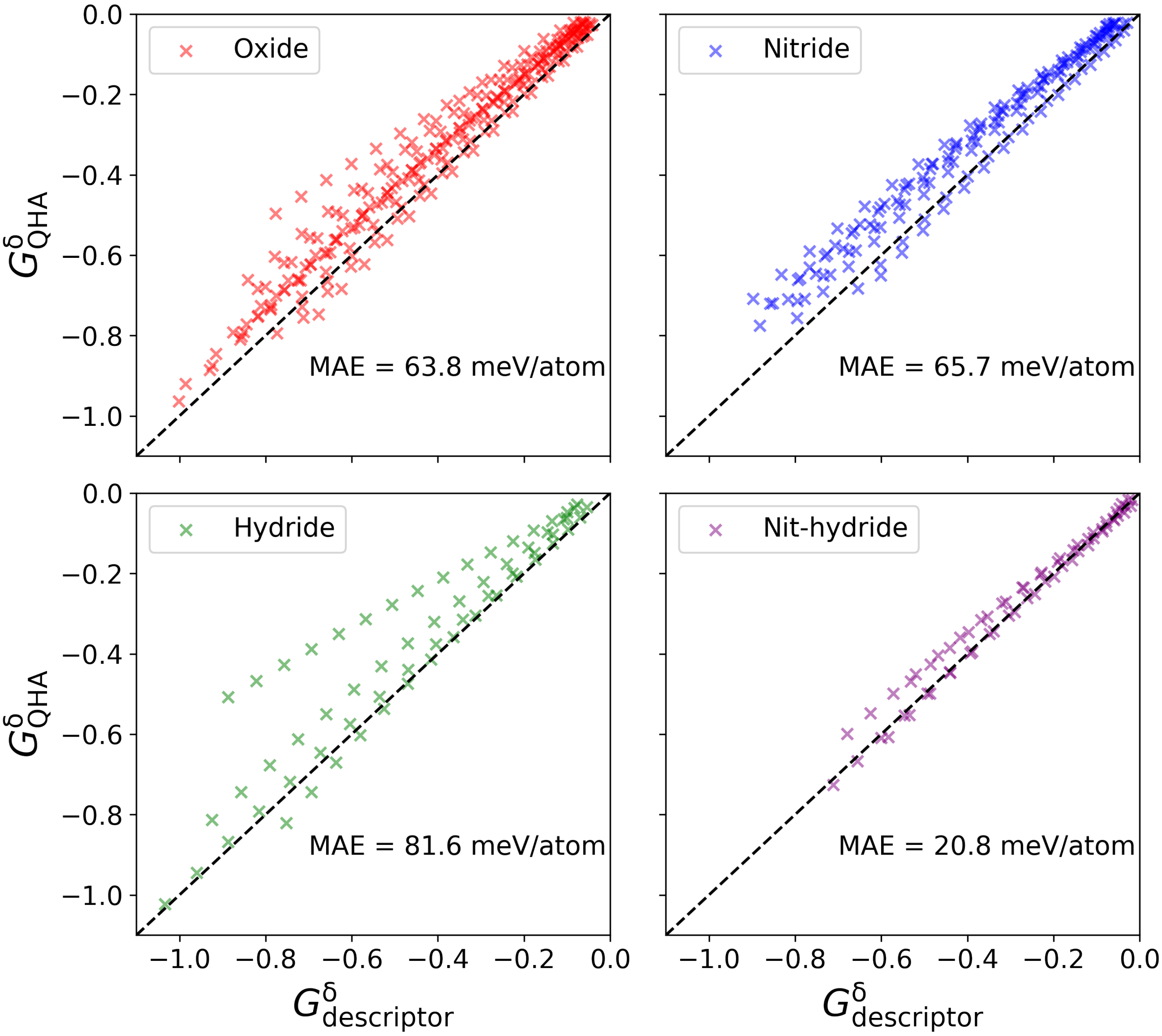

Figure S13: Comparison of Gibbs energy calculated from the physical descriptor ($G^{\delta}_{\text{descriptor}}$) and from the quasi-harmonic approximation (G^{δ}_{QHA}).

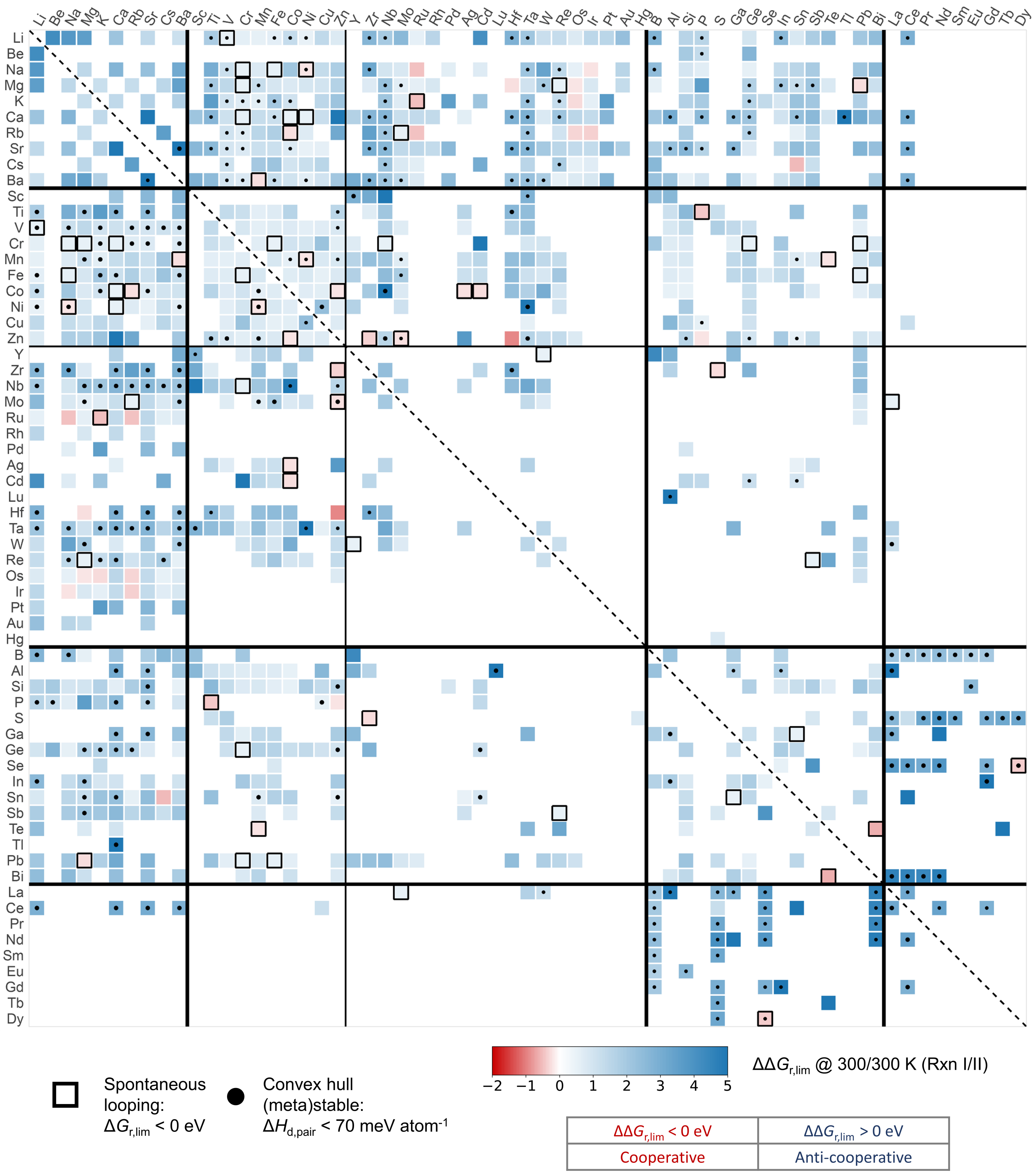

Figure S14: Heatmap of limiting energy differences between bicationic and monocationic pairs for 2-Step H_2O-CL.

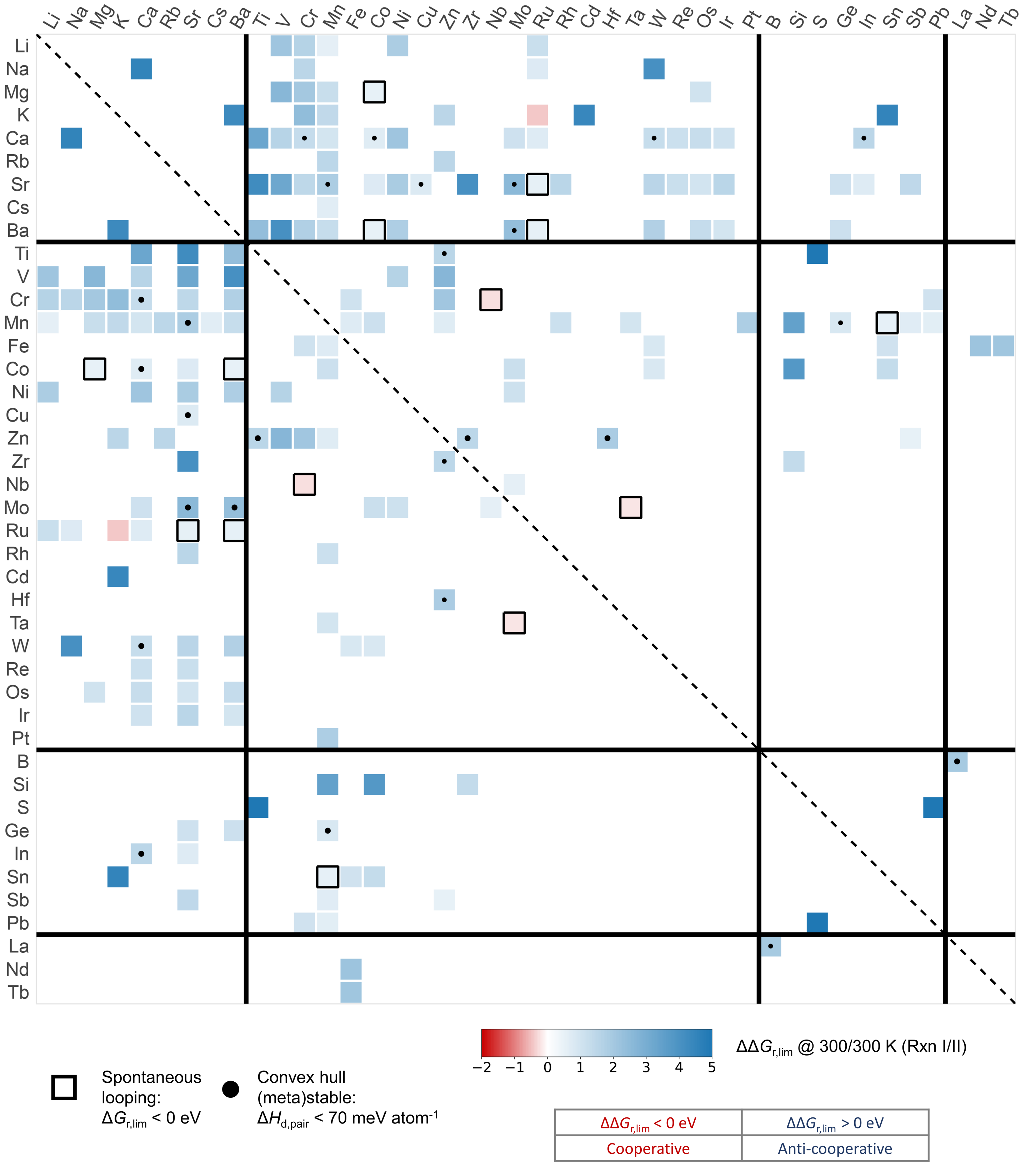

Figure S15: Heatmap of limiting energy differences between bicationic and monocationic pairs for H_2-CL.

References

[1] S. P. Ong, S. Cholia, A. Jain, M. Brafman, D. Gunter, G. Ceder, K. A. Persson, *Computational Materials Science* **2015**, *97* 209.

[2] A. Jain, G. Hautier, S. P. Ong, C. J. Moore, C. C. Fischer, K. A. Persson, G. Ceder, *Physical Review B* **2011**, *84*, 4 045115.

[3] C. J. Bartel, J. R. Rumptz, A. W. Weimer, A. M. Holder, C. B. Musgrave, *ACS applied materials & interfaces* **2019**, *11*, 28 24850.

[4] R. P. Stoffel, C. Wessel, M.-W. Lumey, R. Dronskowski, *Angewandte Chemie International Edition* **2010**, *49*, 31 5242.

[5] A. Togo, L. Chaput, I. Tanaka, G. Hug, *Physical Review B* **2010**, *81*, 17 174301.

[6] A. Togo, I. Tanaka, *Scripta Materialia* **2015**, *108* 1.

[7] G. Kresse, J. Furthmüller, *Physical review B* **1996**, *54*, 16 11169.

[8] G. Kresse, D. Joubert, *Physical review b* **1999**, *59*, 3 1758.

[9] C. J. Bartel, S. L. Millican, A. M. Deml, J. R. Rumptz, W. Tumas, A. W. Weimer, S. Lany, V. Stevanović, C. B. Musgrave, A. M. Holder, *Nature communications* **2018**, *9*, 1 4168.

[10] Y. Zuo, M. Qin, C. Chen, W. Ye, X. Li, J. Luo, S. P. Ong, *Materials Today* **2021**, *51* 126.

[11] J. Wu, Z. Wang, S. Li, S. Niu, Y. Zhang, J. Hu, J. Zhao, P. Xu, *Chemical Communications* **2020**, *56*, 50 6834.

[12] K. Chu, Q.-q. Li, Y.-h. Cheng, Y.-p. Liu, *ACS Applied Materials & Interfaces* **2020**, *12*, 10 11789.

[13] Y. Li, L. Ma, Y. Fu, C. Zhang, Y. Shi, Y. Xu, J. Li, *Journal of Electroanalytical Chemistry* **2022**, *905* 115981.

[14] I. A. Amar, R. Lan, C. T. Petit, S. Tao, *Int. J. Electrochem. Sci* **2015**, *10*, 5 3757.

[15] S. M. AlShehri, J. Ahmed, T. Ahamad, P. Arunachalam, T. Ahmad, A. Khan, *RSC advances* **2017**, *7*, 72 45615.

[16] C. Zhang, H. Guo, Y. Gao, Y. Gong, C. Jin, J. He, *Chemical Physics Letters* **2022**, *793* 139429.

[17] J. Wang, L. Li, H. Tian, Y. Zhang, X. Che, G. Li, *ACS Applied Materials & Interfaces* **2017**, *9*, 8 7100.

[18] J. G. Lee, J. Hwang, H. J. Hwang, O. S. Jeon, J. Jang, O. Kwon, Y. Lee, B. Han, Y.-G. Shul, *Journal of the American Chemical Society* **2016**, *138*, 10 3541.

[19] N. Ahmad, R. Wahab, S. Manoharadas, B. F. Alrayes, F. Alharthi, *Sustainability* **2021**, *13*, 24 13855.

[20] P. Balasubramanian, M. Annalakshmi, S.-M. Chen, T. Sathesh, T.-K. Peng, T. Balamurugan, *ACS Applied Materials & Interfaces* **2018**, *10*, 50 43543.

[21] X.-M. Wang, Y.-N. Zeng, L.-Q. Jiang, Y.-T. Wang, J.-G. Li, L.-L. Kang, R. Ji, D. Gao, F.-P. Wang, Q. Yu, et al., *Industrial Crops and Products* **2022**, *182* 114937.

[22] J. F. Gómez-García, J. A. Mendoza-Nieto, A. Yañez-Aulestia, F. Plascencia-Hernández, H. Pfeiffer, *Fuel Processing Technology* **2020**, *204* 106404.

[23] X. Ren, C. Wei, Y. Sun, X. Liu, F. Meng, X. Meng, S. Sun, S. Xi, Y. Du, Z. Bi, et al., *Advanced Materials* **2020**, *32*, 30 2001292.

[24] S. MSP, M. Hossain, G. Gnanasekaran, Y. S. Mok, et al., *Catalysts* **2019**, *9*, 1 68.

[25] N. Ahmad, F. Alharthi, M. Alam, R. Wahab, S. Manoharadas, B. Alrayes, *Energies* **2021**, *14*, 10 2928.

[26] F. Gao, X. Tang, H. Yi, S. Zhao, W. Zhu, Y. Shi, *Journal of Environmental Sciences* **2020**, *89* 145.

[27] C. Yang, G. Rousse, K. Louise Svane, P. E. Pearce, A. M. Abakumov, M. Deschamps, G. Cibin, A. V. Chadwick, D. A. Dalla Corte, H. Anton Hansen, et al., *Nature communications* **2020**, *11*, 1 1378.

[28] J. Sang, P. Wei, T. Liu, H. Lv, X. Ni, D. Gao, J. Zhang, H. Li, Y. Zang, F. Yang, et al., *Angewandte Chemie* **2022**, *134*, 5 e202114238.

[29] G. K. K. Gunasooriya, J. K. Nørskov, *ACS Energy Letters* **2020**, *5*, 12 3778.

[30] F. Chang, Y. Guan, X. Chang, J. Guo, P. Wang, W. Gao, G. Wu, J. Zheng, X. Li, P. Chen, *Journal of the American Chemical Society* **2018**, *140*, 44 14799.

[31] H. Yan, W. Gao, Q. Wang, Y. Guan, S. Feng, H. Wu, Q. Guo, H. Cao, J. Guo, P. Chen, *The Journal of Physical Chemistry C* **2021**, *125*, 12 6716.
