# Supplementary material for: High‐Throughput Screening of Bicationic Redox Materials for Chemical Looping Ammonia Synthesis
Source: Adv Sci (Weinh). 2022 Jul 24;9(27):2202811. doi: 10.1002/advs.202202811 (PMC9507380; doi:10.1002/advs.202202811)
Supplement: Supplementary file 2 — Supporting Information [file ADVS-9-2202811-s002.html]

Limiting energy differences
